# Does nematic order allow groups of elongated cells to sense electric fields better?

**DOI:** 10.1371/journal.pone.0325800

**Published:** 2025-06-25

**Authors:** Kurmanbek Kaiyrbekov, Brian A. Camley

**Affiliations:** 1 William H. Miller III Department of Physics & Astronomy, Johns Hopkins University, Baltimore, MD, United States; 2 Thomas C. Jenkins Department of Biophysics, Johns Hopkins University, Baltimore, MD, United States; KIST: Korea Institute of Science and Technology, GERMANY

## Abstract

Collective response to external directional cues like electric fields helps guide tissue development, regeneration, and wound healing. In this study we focus on the impact of anisotropy in cell shape and local cell alignment on the collective response to electric fields. We model elongated cells that have a different accuracy sensing the field depending on their orientation with respect to the field. With this framework, we assume cells are better sensors if they can align their long axes perpendicular to the field. Elongated cells often line up with their long axes in the same direction — “nematic order” – does a nematic cell-cell interaction allow groups of cells to share information about their orientation to sense fields more accurately? We use simulations of a simple model to show that if cells orient themselves perpendicular to their average velocity, alignment of a cell’s long axis to its nearest neighbors’ orientation can in some circumstances enhance the directional response to electric fields. We also show that cell-cell adhesion modulates the accuracy of cells in the group.

## Introduction

Electric fields are ubiquitous in the extracellular environments of plants, animals, and humans. They play crucial roles in biological processes such as development, physiology, regeneration, and pathology [1, 2]. Cells, both individually and collectively, follow electric fields; this response is called galvanotaxis or electrotaxis [3]. While many cells involved in wound healing exhibit galvanotaxis, the exact extent and manner of collective cell response to field hinge on factors like cell type, cluster size, electric field strength, and intercellular interactions [3, 4]. For instance, among skin cells, human keratinocytes exhibit a significantly faster response to electric stimuli than human dermal fibroblasts [5, 6]. Additionally, isolated epithelial (MDCK II) cells follow external electric fields less accurately compared to when they are in groups. The accuracy of the electrotactic response of groups of these these epithelial cells increases with group size and is dependent on the presence of cell-cell adhesion [7]; similar results are seen in the MCF10A epithelial cell line [4]. However, evidence suggests that in certain contexts, a reduction in cell adhesion may increase the directedness of cells in an electric field, as demonstrated for primary mouse keratinocytes [8], although the mechanisms behind this phenomenon remain unclear. In this work, we will model the galvanotaxis of clusters of elongated cells to better understand how cell-cell adhesion, cell shape, and cell-cell alignment influence the ability of groups of cells to follow an electric field.

Many types of cells when galvanotaxing tend to align their long axes perpendicularly to the field [3, 6, 9–12]. We hypothesize that this may allow cells to better sense the direction of the electric field – i.e. that a cell’s accuracy at sensing the field direction is better if the cell is elongated perpendicular to the field. Theoretical studies on chemical gradient sensing have shown that elliptical cells oriented orthogonally to the gradient may estimate its direction with higher accuracy [13]. Similarly, theoretical investigations by our group into galvanotactic sensing suggest that, under certain conditions, cells may also exhibit enhanced sensing precision for the field’s angle when oriented perpendicular to the electric field [14]. Our current understanding of galvanotaxis suggests it involves the redistribution of proteins or other sensing molecules along the cell membrane [15]. Cells may estimate the field direction based on the angular distribution of these molecules [16]. For anisotropic, elliptical cells, this distribution depends on orientation: the longer axis provides a broader surface area for protein redistribution compared to the shorter axis and this anisotropy may manifest in orientation-dependent differences in sensing precision. However, it is not straightforward for single cells to *benefit* from this additional precision. For a single cell to gain any information from anisotropic sensing, it must turn its long axis perpendicular to the field orientation – requiring some initial knowledge about the field orientation, or adaptation of orientation based on past sensed gradient orientation [17]. One possibility is that *groups* of elongated cells may benefit more from anisotropic sensing, because groups of cells can share information by aligning their long axes with each other. This alignment of long axes or “nematic ordering” occurs in confluent elongated cells even in the absence of an applied field [18–22]. We want to know under what circumstances nematic alignment allows cells to reliably share information and pick an orientation perpendicular to the applied field, and under what circumstances this fails.

We develop a simple model to explore how groups of elongated cells with anisotropic precision sense electric fields. We hypothesize that cells use their past motion to set the orientation of their long axis, ensuring that a cell’s long axis eventually becomes aligned perpendicular to the field direction. Unsurprisingly, if cells are better sensors when perpendicular to the field, the alignment of the long axis improves group galvanotaxis. More importantly, we show that local nematic interactions between cells generally enhances the directionality of clusters, though this depends on the strength of the cell-cell interactions. Lastly, we demonstrate that strong cell-cell adhesion amplifies the directional response to electric fields, while also identifying mechanisms that may disrupt this response, offering potential explanations for conflicting experimental results on the role of adhesion in collective galvanotaxis.

## Model

**Fig 1:**
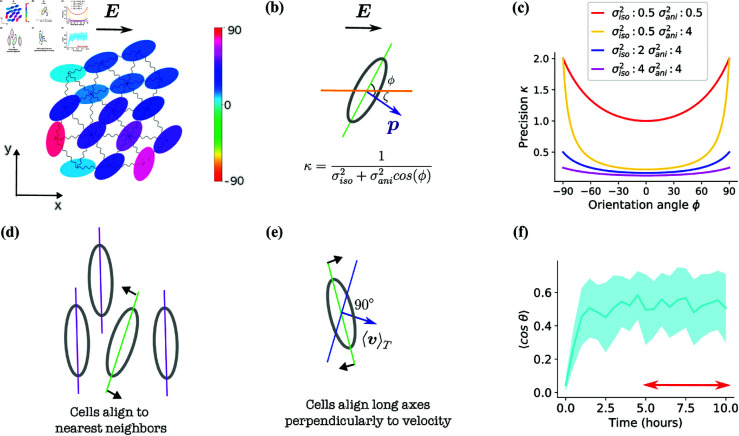
Description of cell parameters and simulation model. **(a)** The depicted snapshot illustrates a simulation box, showcasing 16 interacting cells interconnected by springs. The electric field within this model is oriented along the positive x-axis. Each cell is colored by the angle formed between the x-axis and the cell’s major axis. **(b)** A cell exposed to field **E** estimates the direction of the field and polarizes (changes its polarity direction ζ) towards its estimate. A cell’s precision κ in detecting the electric field direction, depends on its orientation angle ϕ, where ϕ is the shortest angle between the cell’s longitudinal axis (green line) and the x-axis (orange line). With this definition, ϕ will be between $-90^{\circ }$ and $+90^{\circ }$. **(c)** Cell precision κ as a function of cell orientation for different values of intrinsic ($\sigma_{\text{iso}}^{2}$) and anisotropic ($\sigma_{\text{ani}}^{2}$) variances. Cells oriented horizontally (ϕ=0) have the lowest precision and cells with vertical orientation ($\phi =90^{\circ }$) have the highest precision. **(d)** A diagram illustrating the tendency of a cell, with its long axis represented by a green line, to align with neighboring cells, whose long axes are depicted with purple lines. **(e)** A diagram depicting the tendency of a cell to align its orientation, illustrated by a green line, perpendicularly to the cell’s average velocity. The orientation orthogonal to the velocity is represented by a blue line. **(f)** The graph presents the temporal evolution of the average directionality of cell groups across 40 simulations, with shaded areas representing the standard deviations calculated over these simulations. Each simulation featured 64 cells. The red arrow spans the final 5 hours and we report the mean over this period as the final steady state directionality.

We use a two-dimensional self-propelled particle model to describe cell behavior in the presence of a constant electric field $E=E\hat{x}$ (Fig 1a), where $\hat{x}$ is the unit vector in the x-direction. We model elongated cells as particles characterized by positions $r^{i}=(x^{i},y^{i})$ and the orientation of the long axis $\phi^{i}$ for the $i^{\text{th}}$ cell (Fig 1b). Each cell will also have a polarity direction $\zeta^{i}$ – the direction the cell is propelling itself in. Cells exhibit an orientation-dependent accuracy in sensing the field, are interconnected through spring-like adhesions, align themselves perpendicular to their velocity, and orient their long axes in alignment with those of their neighbors (Fig 1a–e).

### Translational motion

Cells have an internal biochemical polarity that sets the direction of their motion. This polarity arises due to asymmetry between the back of the cell where myosin contractions pull the rear, and front where filopodia and/or lamellipodia extend the cell frontier [23]. The polarity of cell *i* $p^{i}=(p_{x}^{i},p_{y}^{i})=p_{0}(\cos\zeta^{i},\sin\zeta^{i})$ is the velocity that the cell would have in the absence of interactions with other cells. The polarity vector has a magnitude of $p_{0}=1\;\mu \text{m/min}$ (equivalent to 60 μm / h), roughly consistent with typical single-cell speeds [24, 25]. We evolve cell positions according to over-damped Newtonian equations of motion:

$$\frac{\partial r^{i}}{\partial t}=p^{i}+\sum_{j\underset{n}{\sim }i}F^{ij}$$ (1)

where $F^{ij}$ is an inter-cellular force (e.g. adhesion and volume exclusion) by cell *j* on cell *i*, and the sum is over the neighbors that are within the interaction cutoff distance *r*_*c*_ from the cell *i* (the interacting neighbors *j* of cell *i* are denoted as $j\underset{n}{\sim }i$). If the distance between two interacting cells exceeds the interaction cutoff distance *r*_*c*_ they cease to interact – modeling a disconnection of cell-cell adhesion. The force arises due to neighboring cells interacting with a harmonic spring:

$$U^{ij}=\frac{1}{2}k(r^{ij}-r_{eq}{)}^{2}$$ (2)

where $r^{ij}=|r^{i}-r^{j}|$ is the distance between cells *i* and *j*, and *r*_*eq*_ is a parameter that sets an equilibrium distance between cells. The force exerted by cell *j* on cell *i* is given by $F^{ij}=-\nabla_{i}U^{ij}$. Here, the spring constant k, which we can think of as setting the cell-cell adhesion strength, is expressed in units of 1/time – implicitly this means that we have absorbed a friction coefficient into the value of k instead of writing it in Eq 1. We note that the spring interaction does not depend on the particle orientation – the effect of the orientation is solely on the the cell’s ability to sense the field direction and on cell-cell orientational alignment.

### Cell polarity and field sensing

We assume that cells estimate the field direction and then polarize in the direction of their estimate. (This neglects any dynamics of reaching this estimated direction [26, 27].) We also assume that precision of a cell’s estimate depends on its orientation such that it has highest precision when oriented orthogonally to electric field, and lowest precision if its long axis is parallel to the field. We model this estimation process as the cell drawing its estimated direction $\zeta^{i}$ from a von Mises distribution centered around the true electric field angle with respect to the x-axis (which is 0), with a width controlled by the precision $\kappa^{i}$ which depends on cell *i*’s orientation, i.e. $\zeta^{i}\sim VM(0,\kappa^{i})$. The von Mises distribution is a generalization of the normal distribution to orientations [28], and has a form $p(\zeta )\sim e^{\kappa \cos(\zeta )}$ – i.e. in the limit of large κ it corresponds to a normal with variance $\sigma^{2}=1/\kappa$ – and we will often refer to errors in terms of $\sigma^{2}$. The cell polarizes in the direction of its estimate $\zeta^{i}$:

$$p^{i}=p_{0}\cos(\zeta^{i})\hat{x}+p_{0}\sin(\zeta^{i})\hat{y}$$ (3)

(see Fig 1b).

The precision of the estimate $\kappa^{i}$ is orientation-dependent. We hypothesize that cells exhibit the highest precision when perpendicular to the electric field $E=E\hat{x}$ (vertical orientation) and lowest precision in parallel (horizontal) orientation:

$$\kappa^{i}=\frac{1}{\sigma_{\text{iso}}^{2}+\sigma_{\text{ani}}^{2}\cos\phi^{i}}$$ (4)

where the cell orientation $\phi^{i}\in [-\pi /2,\pi /2]$ is the smallest angle between the cell’s long axis and the x-axis (Fig 1b). Hence, a cell *i* in vertical orientation ($\phi^{i}=\pm \pi /2$) would have $\kappa^{i}=1/\sigma_{\text{iso}}^{2}$ – or an angular error of $\sigma_{\text{iso}}^{2}$ – while in horizontal alignment ($\phi^{i}=0$) would have a precision of $\kappa^{i}=1/(\sigma_{\text{iso}}^{2}\;+\;\sigma_{\text{ani}}^{2})$ – or an angular error of $\sigma_{\text{iso}}^{2}\;+\;\sigma_{\text{ani}}^{2}$. The $\sigma_{\text{iso}}^{2}$ is the orientation-independent intrinsic variance – the baseline accuracy in estimation – and $\sigma_{\text{ani}}^{2}$ characterizes the additional variance stemming from the anisotropy of cell shape. The orientation dependence of precision is most pronounced when the difference between values of $\sigma_{\text{iso}}$ and $\sigma_{\text{ani}}$ is substantial (see Fig 1c).

Cells can make an estimation of the electric field multiple times over our simulation. How often should we expect this estimation to happen? Galvanotaxis – in our best current understanding – requires redistribution of proteins or other sensor molecules on the cell membrane [15, 16]. Membrane proteins on a cell of radius *R* redistribute via diffusion in a characteristic time $\tau_{\text{forget}}=R^{2}/D$, where *D* is the protein diffusion constant. Averaging time-correlated estimates of the electric field direction for a duration less than $\tau_{\text{forget}}$ does not improve accuracy [16]. Therefore, we assume that cells estimate field direction every $\tau_{\text{forget}}$ minutes and keep the polarity to be constant until a new estimate is made. We estimate $\tau_{\text{forget}}$ to be on the order of 10 minutes [15, 16], though this depends on the cell type and properties of the sensor protein.

### Cell reorientation

Elongated cells in confluence exhibit a tendency to align with neighboring cells, resulting in the formation of a local nematic order [20–22, 29] (see Fig 1d). Additionally, when exposed to an electric field, many cell types, even in isolation, will follow the field but orient themselves orthogonally to it [3, 6, 10–12]. In other words, on average, cells align their long axis perpendicularly to their velocity vector (depicted in Fig 1e). We incorporate these tendencies into the equations of motion for $\phi^{i}$, the orientation of the long axis of cell *i*:

$$\frac{\partial \phi^{i}}{\partial t}=-\;\Omega_{v}\sin(2[\phi^{i}-(\alpha_{\langle v^{i}\rangle_{T}}+\pi /2)])\;\;\;-\;\Omega_{n}\sum_{j\underset{n}{\sim }i}\sin(2[\phi^{i}-\phi^{j}])+\sqrt{2D_{r}}\xi_{r}(t)$$ (5)

The first term in Eq 5 represents the cell’s orientation tending to rotate to be π/2 away from the direction of the time-averaged velocity $\alpha_{\langle v^{i}\rangle_{T}}$, with a rate of alignment $\Omega_{v}$. Here, we define $\alpha_{\langle v^{i}\rangle_{T}}$ as the angle between $\langle v^{i}\rangle_{T}$ and horizontal axis, where $\langle v^{i}\rangle_{T}$ is the velocity averaged over past period *T*, i.e. if $\partial {𝐫}^{i}/\partial t$ is the instantaneous velocity of the cell *i* then $\langle v^{i}\rangle_{T}=(1/T)\int_{t-T}^{t}(\partial {𝐫}^{i}/\partial t)dt$.

Why do we choose the cell to orient perpendicular to the time-averaged velocity – or equivalently, to the displacement of the cell over a time *T*? First, experiments observe that galvanotaxing cells often are oriented with long axis perpendicular to the field [3]. As we have emphasized above, and discussed systematically by [17], for cells to benefit from anisotropic sensing, the cell’s orientation must depend on its past measurement of the field orientation. The displacement of the cell over some time is a natural first representation of the cell’s past measurements, and we think a cell could compute it simply. For instance, integrins are anchored to the substrate, so focal adhesions have an anchor point to the substrate while the cell moves. This means that the cell’s displacement directly affects the relative motion of focal adhesions and the cell’s interior since actin filaments connect to these focal adhesions and reorganize to exert strong contractile forces, guiding the cell’s direction of motion, internal organization, and overall orientation. This interplay also creates a feedback loop: forces generated by the cytoskeleton feed back into focal adhesions, further modulating the migration direction. The information from pulling and anchoring dynamics of focal adhesions could be used by cells to estimate the migration direction and align orthogonally. For this reason, other groups have assumed that cells develop polarity in a direction that reflects displacement over a timescale *T* [30, 31] – our approach here is somewhat similar, but separates out cell orientation instead of polarity. The choice of modeling cells expanding perpendicular to their velocity has been used earlier both in our work on single-cell galvanotaxis [26] and earlier models of cell motility [32] and self-propelled deformable particles [33, 34]. These models were largely proposed on the grounds of symmetry and simplicity but these effective models may also arise from more detailed biochemical modeling of coupling between cell polarity and cell shape or more general reaction-diffusion models [34–37].

Within our model, the choice of the timescale *T* will be important, because the instantaneous velocity of a cell is highly variable, influenced by the current estimate of the electric field direction and interactions with neighboring cells. As the timescale *T* is increased, cells utilize larger displacements to estimate migration direction and $\langle v^{i}\rangle_{T}$ will be an increasingly reliable indicator of the net migration direction. If we assume that cells employ the reorientation mechanism proposed in our model, we could expect a cell-type dependent variation in averaging times *T* since different cells have different timescales for reaching steady-state orientations during electrotaxis [3, 38]. In our study we conduct simulations for various *T* to establish value that results in meaningfully reliable estimations for our parameter settings.

The second term in Eq 5 nematically aligns a cell’s long axis with that of its neighbors *j* with a rate of alignment $\Omega_{n}$ – i.e. it ensures that cells’ long axes are either parallel or antiparallel. Finally, *D*_*r*_ is the rotational diffusion coefficient which describes spontaneous reorientation of the cell, and $\xi_{r}$ is a Langevin noise term with Gaussian probability distribution that has a zero mean $\langle \xi_{r}(t)\rangle =0$ and time correlation $\langle \xi_{r}(t_{1})\xi_{r}(t_{2})\rangle =\delta (t_{1}-t_{2})$.

In this paper, we always simulate motion in the presence of an electric field. However, we note that in absence of electric field, corresponding to complete uncertainty about field orientation $\kappa^{i}\to 0$, cells would sample polarization directions uniformly. In this case, a single cell would essentially be undergoing a random walk, with a new orientation chosen every $\tau_{\text{forget}}$.

## Results

The key parameters that we will measure in our simulations are cell directionalities – the average cosine of the angle between the cell velocity and the field ⟨cosθ⟩ – and cell speeds ⟨v⟩. To be consistent with experiments which measure cell velocities over a fixed time window [3, 4, 7, 8, 12], we also compute individual cell velocities at intervals of 30 minutes. For instance, if the displacement of cell *i* is $\Delta {𝐫}^{i}=\Delta x^{i}\hat{x}\;+\;\Delta y^{i}\hat{y}$ during 30 minutes, then the cell velocity is $\Delta {𝐫}^{i}/30\text{min}=\langle v^{i}\rangle_{T=30\text{min}}=|\langle v^{i}\rangle_{T=30\text{min}}|(\cos\theta^{i}\hat{x}\;+\;\sin\theta^{i}\hat{y})$, where $\left|\langle v^{i}\rangle_{T=30\text{min}}\right|$ is the magnitude of the measured velocity.

We conduct 𝒮=40 simulations of 10 hours for each parameter set. Initially, cells begin at random orientations – so they cannot benefit from the increased accuracy when pointed perpendicular to the field. As the simulation evolves, cells estimate the direction of the electric field, update their polarities accordingly, and migrate toward this estimated direction. This movement is further influenced by the forces exerted by neighboring cells. Simultaneously, the cells adjust their orientations by aligning themselves perpendicularly to their averaged velocity and to nearest neighbors. Then, they update their estimates of field direction, and this cycle continues (Movie 1). Measuring the directionality averaged across cells and across simulations, we observe that directionality increases over the initial 3-4 hours and then subsequently reaches a steady state (Fig 1f). During this time, cells become more “vertically” aligned. Through the rest of the paper, we will report the velocity and directionality averaged over the final 5 hours of simulation to characterize their steady-state values (red arrow in Fig 1f).

### Sufficient anisotropy in sensing accuracy and alignment to average velocity is necessary to see benefits of favorable alignment

**Fig 2:**
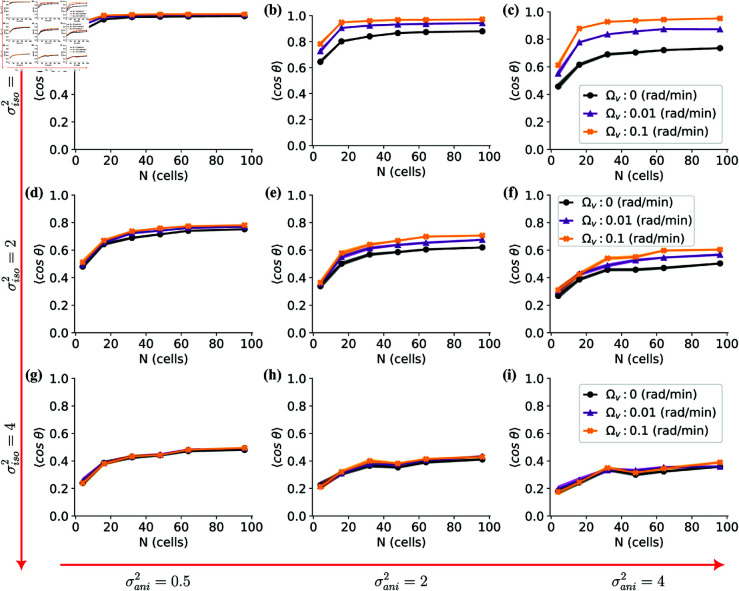
The average directionality ⟨cosθ⟩ for various combinations of isotropic $\sigma_{\text{iso}}^{2}$ and anisotropic $\sigma_{\text{ani}}^{2}$ variances and alignment rates to velocity. Isotropic component ($\sigma_{\text{iso}}^{2}$) changes across rows (top to bottom) and anisotropic component ($\sigma_{\text{ani}}^{2}$) across rows (left to right) with specific values shown at right side and bottom of the figure (i.e. the panel (f) show directionalities for $\sigma_{\text{iso}}^{2}=2,\sigma_{\text{ani}}^{2}=4$ ). The averages are over 40 simulations at the interaction strength of *k* = 0.2 ${\text{min}}^{-1}$. For each simulation the reported directionality is the steady state average over the final 5 hours of simulation as shown in Fig 1f. Results for different values of alignment rates to average velocity are color coded $\Omega_{v}=0$ rad/min (black), $\Omega_{v}=0.01$ rad/min (purple), and $\Omega_{v}=0.1$ rad/min (orange). The averaging time *T* for velocity is set to 1h, and the number of cells *N* was set to 4, 16, 32, 48, 64, and 96. The shaded areas represent standard errors, although they may not be easily discernible due to their small size.

We hypothesized that cells are more accurate sensors when perpendicular to the field (“vertical”). To what extent does this assumption enhance cells’ directional motion in a cluster? There are two key ingredients for sensing anisotropy to improve cluster accuracy: 1) the difference between sensing in the vertical orientation and the horizontal orientation must be large, and 2) cells must manage to reach the vertical orientation. Within our model, the term that drives cells toward the vertical orientation is the alignment perpendicular to velocity, controlled by $\Omega_{v}$. We modulate the rate of alignment to the average velocity ($\Omega_{v}$) across varying cell cluster sizes (*N*) for varying $\sigma_{\text{iso}}$ and $\sigma_{\text{ani}}$ in Fig 2. We initially set the alignment rate to nearest neighbors ($\Omega_{n}$) to zero to isolate the effect of $\Omega_{v}$, and will study nematic alignment effects later. From Fig 2, we see initially that an increase in group size (*N*) generally correlates with an improvement in directionality, regardless of the combinations of variances and alignment rates.

In Fig 2, we observe that the alignment rate to velocity $\Omega_{v}$ – which controls to what extent the cell has a vertical orientation – only has a relevant effect in the upper-right four graphs, which are the cases where isotropic error $\sigma_{\text{iso}}$ is relatively low and anisotropic $\sigma_{\text{ani}}$ error is relatively high. This is what we would expect. If the anisotropic variance is large while the isotropic error small, cells experience reduced precision if they significantly deviate from the favorable vertical orientation. Consequently, the advantages of faster alignment to average velocity become pronounced (see Fig 2b and c). By contrast, if cells have near-perfect estimates of the field direction independent of orientation (e.g. panel a, $\sigma_{\text{iso}}^{2}=0.5$ and $\sigma_{\text{ani}}^{2}=0.5$), then the cells within groups have near-perfect directionality independent of rates of alignment. Similarly, if cells’ error is dominated by the isotropic component, e.g. $\sigma_{\text{iso}}^{2}=2$, cells experience a reduction in precision for all orientations, leading to an overall low level of directionality and no strong dependence on $\Omega_{v}$(Fig 2d–f).

For the remainder of this paper, we adopt $\sigma_{\text{iso}}^{2}=2$ and $\sigma_{\text{ani}}^{2}=4$ as our default parameters – panel f of Fig 2, S1 Movie. We selected these values because they yield a directionality trend, with respect to the number of cells, that is roughly consistent with the findings of Li *et al*. [7] for MDCK cells. The ratio of variance between the horizontal ($\sigma_{\text{iso}}^{2}\;+\;\sigma_{\text{ani}}^{2}$) and vertical ($\sigma_{\text{iso}}^{2}$) orientations, which is $\frac{4+2}{2}=3$ are roughly consistent with plausible numbers motivated by studies of chemotaxis [13], where the ratio of variance between the horizontal direction and vertical direction is ≈4 for a cell with aspect ratio 2. Comparable variations in sensing accuracy are also observed in the results on galvanotactic sensing [14], though these depend on both the field’s magnitude and the cell’s interpretation of it.

Why does directionality improve with increasing *N*? Initially, this may not be surprising – previous models of collective gradient sensing often found that the directionality of the cluster center of mass increases with cell number [39]. The cluster center of mass will almost always have a decreased noise as the number of cells increases simply from the law of large numbers because the center of mass motion reflects the average of many noisy motions of individual cells (see, e.g. [40]; we repeat the basic argument in our S1 Appendix later). However, the directionalities we plot in Fig 2 are for *single* cells – not the center of mass. Why should directionality increase here? We argue this arises from cell-cell adhesion in our model. Even though the motion of the center of mass is independent of adhesion strength *k*, the spring strength *k* controls the degree to which an individual cell follows the center of mass within a given time. In the limit of very large *k*, the cell cluster is essentially a rigid body – each cell perfectly follows the center of mass. On the other hand, for very small values of *k*, each cell behaves almost as an independent unit, only weakly tracking the group’s center of mass. Hence, we expect minimal benefits from increasing cluster size in the case of weak adhesion but much stronger *N* dependence in the case of strong adhesion. We repeat the simulations of Fig 2 with very weak (*k* = 0.05 ${\text{min}}^{-1}$) and strong (*k* = 1 ${\text{min}}^{-1}$) adhesion strengths. Consistent with our expectations, directedness improves as cell groups get larger for strong adhesive forces (S1 Fig, S2 Movie) while there are only small benefits of increasing cell numbers for weakly interacting cells (S2 Fig, S3 Movie). The essential role of cell-cell adhesion to gain a benefit in group galvanotaxis is consistent with experimental measurements showing E-cadherin is necessary for MDCK group galvanotaxis [7]. We will explore the role of adhesion strength in more detail later.

### Increasing velocity averaging time improves vertical alignment of cells and directionality

Because cells align perpendicular to their average velocity, and cells are better sensors when perpendicular to the field, there is a natural feedback between orientation and velocity. As cells follow the applied field more accurately, their average velocity becomes more aligned to the electric field. This, in turn, means the cells become increasingly orthogonal to the field, improving their ability to sense the field. Because the instantaneous velocity of a cell will reflect both its self-propulsion and forces from neighboring cells, it will fluctuate around the true direction of the field. We expect that the velocity averaged over some time *T* will thus have less variability and more reliably point toward the field – so we expect that increasing the averaging time will increase the cell directionality as well as making the cells increasingly vertically-oriented.

**Fig 3:**
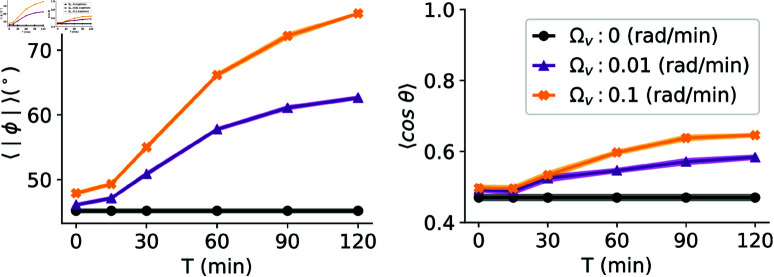
Cell orientations and directionalities as a function of velocity averaging time. In the left column, the absolute value of the cell orientation angle is presented, while the right column displays the directionality. The reported values represent averages across 40 simulations of 64 cells conducted with an interaction strength of *k* = 0.2 ${\text{min}}^{-1}$ and $\sigma_{\text{iso}}^{2}=2\;\&\;\sigma_{\text{ani}}^{2}=4$. The shaded areas represent standard errors of the mean.

Running simulations while varying the averaging time *T*, we do see that cells become increasingly vertical and directional as *T* increases (Fig 3). This dependence on averaging time, naturally, only happens if $\Omega_{v}>0$, i.e. that cells orient perpendicular to their averaged velocity. The simulations of Fig 3 are with our default adhesion level of *k* = 0.2 ${\text{min}}^{-1}$. Further increases in *T* would dampen fluctuations even more, and in the limit of large *T*, the $\alpha_{\langle v^{i}\rangle_{T}}$ would align with the electric field. Consequently, cells would have information about the true direction of the electric field and would orient orthogonally to it. This would cause cells to assume almost vertical orientations at higher alignment rates $\Omega_{v}$, and in this regime, directionalities would plateau. However, we believe that biologically averaging over *T*>1 hour is unlikely, as cells have been shown to respond to electric fields on a timescale of approximately 15 minutes [3], depending on the cell type.

Cells connected with stiffer springs (*k* = 1 ${\text{min}}^{-1}$) will have faster dynamics of cell-cell interactions, and we see pronounced improvement in alignment and directionality even for smaller averaging times (see S3b Fig). Weakly interacting cells move more like individual units and while increasing averaging times improves alignment a little bit it does not relevantly improve the directedness of cells (S3a Fig). This suggests that the relevance of the averaging time is to integrate over fluctuations of cell position due to relative motion from one cell to another, so that the averaged velocity better reflects the cluster center of mass motion.

### Nematic alignment to neighbors improves vertical alignment and directedness

**Fig 4:**
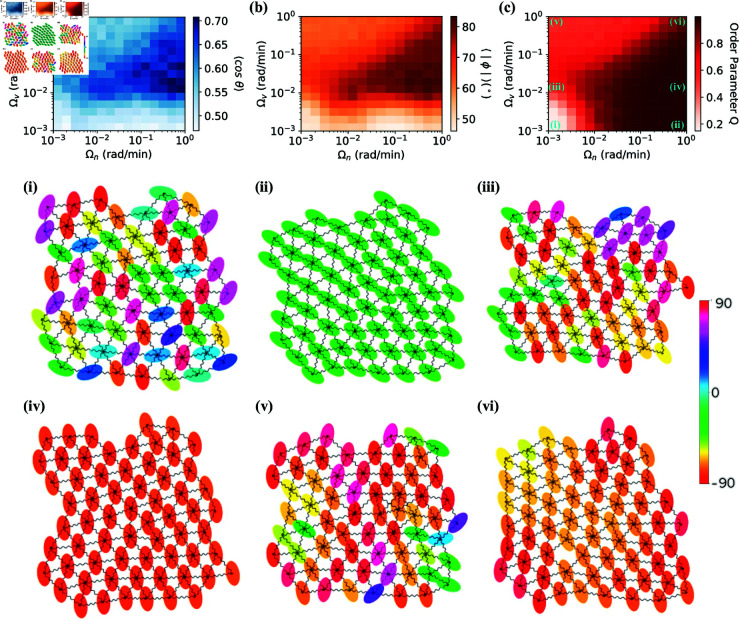
Effect of rates of alignment to velocity ($\Omega_{v}$) and neighbors ($\Omega_{n}$) on directionality (a), absolute value of orientation angle (b), and order parameter (c). Each grid value represents an average result over 40 simulations conducted with 64 cells at the interaction strength of k = 0.2 ${\text{min}}^{-1}$ with an averaging time *T* = 1 h with colorbars indicating corresponding numeric values. Example simulation snapshots for alignment rate tuples of **(i)**, $\Omega_{v}=0.001$ rad/min, $\Omega_{n}=0.001$ rad/min; **(ii)**, $\Omega_{v}=0.001$ rad/min, $\Omega_{n}=1$ rad/min; **(iii)**, $\Omega_{v}=0.012$ rad/min, $\Omega_{n}=0.001$ rad/min; **(iv)**, $\Omega_{v}=0.012$ rad/min, $\Omega_{n}=1$ rad/min; **(v)**, $\Omega_{v}=1$ rad/min, $\Omega_{n}=0.001$ rad/min; **(vi)**, $\Omega_{v}=1$ rad/min, $\Omega_{n}=1$ rad/min, also shown in panel **c**. Cells are colored according to their orientation shown on the colorbar. For all simulations $\sigma_{\text{iso}}^{2}=2$ and $\sigma_{\text{ani}}^{2}=4$.

Following experimental motivation [18, 21, 41], we have assumed that our cells’ long axes have a nematic alignment controlled by $\Omega_{n}$. Can cells use this alignment to work together to align themselves perpendicular to the field, and increase directionality? We simulate cluster migration over a broad spectrum of alignment rates to nearest neighbors $\Omega_{n}$ and average velocity $\Omega_{v}$ in Fig 4. In addition to directionality ⟨cosθ⟩ and extent of vertical alignment of cells ⟨|ϕ|⟩, we quantify the overall alignment of group of cells using the nematic order parameter *Q* [18, 41]

$$Q=\sqrt{\langle \cos2\phi \rangle^{2}+\langle \sin2\phi \rangle^{2}}$$ (6)

where ϕ represents the angle of cell orientation, and the averaging is performed across the cell population. *Q* = 1 means the long axes of the cells are perfectly aligned – but not necessarily vertically aligned – while *Q* = 0 if ϕ is uniformly distributed.

The cell directionality ⟨cosθ⟩ depends on both alignment to velocity $\Omega_{v}$ and alignment to neighbors $\Omega_{n}$ (Fig 4a). Directionality is maximal when alignment to velocity is in our intermediate range ($\Omega_{v}\sim 10^{-2}-10^{-1}$ rad/min) but alignment to neighbors is high ($\Omega_{n}\sim 10^{-1}-10^{0}$ rad/min). The directionality largely reflects the degree to which cells successfully reach a vertical orientation (Fig 4b). We know that in the absence of any alignment to velocity $\Omega_{v}\to 0$, long axes of cells are essentially randomly oriented relative to the field. Consistent with this idea, we see our lowest directionality and lowest ⟨|ϕ|⟩ at low $\Omega_{v}\sim 10^{-3}$ rad/min. Increasing alignment to neighbors while keeping $\Omega_{v}$ low does make cells line up nematically (Fig 4c plots *Q*, and the difference is dramatic in comparing points i and ii). However, increasing $\Omega_{n}$ with low $\Omega_{v}$ fails to induce the vertical alignment necessary to significantly improve directionality (Fig 4b).

There are two key features in Fig 4a that we want to highlight. First, at sufficiently large $\Omega_{v}$, increasing alignment to neighbors ($\Omega_{n}$) enhances directionality. This suggests that cells effectively share information through their nematic alignment. Secondly, the dependence of directionality on alignment to velocity ($\Omega_{v}$) is not monotonic. At low $\Omega_{v}$, cells fail to orient relative to the field and gain no significant directionality benefit. However, at excessively high values of $\Omega_{v}$, directionality decreases again. There are two complementary factors driving this phenomenon. First, as $\Omega_{v}$ increases, cells rapidly align their long axis to be exactly perpendicular to their own averaged velocity $\alpha_{\langle v^{i}\rangle_{T}}\;+\;\pi /2$ (see S6b Fig), neglecting information from their neighbors. Second, even though cells align well to the perpendicular direction of their own averaged velocity, the $\alpha_{\langle v^{i}\rangle_{T}}\;+\;\pi /2$ are themselves quite noisy estimates of the angle perpendicular to the electric field (S6c Fig). Our picture of the non-monotonicity of directionality on $\Omega_{v}$ essentially depends on the size of the terms in Eq 5. At small $\Omega_{v}$, the cell’s orientation is dominated by rotational noise and cell-cell alignment, and can have no correlation to the field direction – leading to low directionality. At intermediate $\Omega_{v}$, though, there is an effective balance between the alignment to velocity and alignment to neighbor orientations that allows a cell to incorporate information from its neighbors about the “correct” orientation – allowing the cell to benefit from averaging out the noisiness of multiple cells’ $\alpha_{\langle v^{i}\rangle_{T}}$. At very large $\Omega_{v}$, the velocity alignment term in Eq 5 dominates all other terms, and cell *i*’s orientation precisely follows its own $\alpha_{\langle v^{i}\rangle_{T}}$ – but because $\alpha_{\langle v^{i}\rangle_{T}}$ is noisy, this leads to a lower directionality than the case with intermediate $\Omega_{v}$.

We can probe the origin of this nonmonotonicity further by changing $\sigma_{\text{iso}}^{2}$ and $\sigma_{\text{ani}}^{2}$ in two different ways. If we increase the degree of anisotropy by raising $\sigma_{\text{ani}}^{2}$ to 6 while keeping $\sigma_{\text{iso}}^{2}=2$, this does not alter the patterns in directionality, alignment, and order (S7a-c Fig). With this change of parameters, we have only made the cells’ errors in sensing larger – so we expect that the estimates of the ideal cell orientation $\alpha_{\langle v^{i}\rangle_{T}}\;+\;\pi /2$ will also become noisier, so we expect to still see the non-monotonic dependence on $\Omega_{v}$. Conversely, if we maintain $\sigma_{\text{ani}}^{2}=4$ and decrease $\sigma_{\text{iso}}^{2}$ to 0.5, the accuracy of cells in *any* orientation significantly improves. This makes $\alpha_{\langle v^{i}\rangle_{T}}\;+\;\pi /2$ a more reliable estimate of the favorable vertical orientation – so cells with large $\Omega_{v}$, who are only using their own $\alpha_{\langle v^{i}\rangle_{T}}$ and not sharing information, may still reach near-optimal orientations. In this case, the non-monotonic dependence on $\Omega_{v}$ is mostly suppressed (S7d-f Fig). We argue the key requirement for non-monotonic behavior to occur is that a single cell’s $\alpha_{\langle v^{i}\rangle_{T}}$ is noisy enough that the cell deviates significantly from the ideal perpendicular orientation. This will occur at sufficiently large $\sigma_{\text{iso}}^{2}$ and $\sigma_{\text{ani}}^{2}$.

Our key trends are somewhat robust to varying degrees of cell adhesiveness. Whether considering cells with weak adhesion (*k* = 0.05 ${\text{min}}^{-1}$, S4 Fig) or those bound by stronger forces modeled as taut springs (*k* = 1 ${\text{min}}^{-1}$, S5 Fig), the influence of $\Omega_{v}$ on vertical alignment is clear. In both scenarios, low $\Omega_{v}$ values fail to produce significant vertical alignment across a broad range of alignment rates to neighbors $\Omega_{n}$. However, for cells interconnected by taut springs, the non-monotonic trend is weaker – rapid alignment at large $\Omega_{v}$ doesn’t greatly impede directionality. This suppression of the nonmonotonic trend in $\Omega_{v}$ at high cell-cell adhesion is consistent with our picture above for the origin of nonmonotonicity – large cell-cell adhesion strength decreases the noise of the time-averaged velocity, because each cell is more aligned with the cluster’s center of mass motion.

### Cell-cell adhesion is crucial for robust directedness of cells

We have noted earlier in this paper that cell-cell adhesion may play a role in controlling directionality of collective galvanotaxis. What do experiments suggest? Research from Min Zhao’s group demonstrated that disrupting E-cadherin junctions in MDCK I cells with the monoclonal antibody DECMA-1 leads to increased cell speeds but diminished galvanotactic directionality [7]. Similarly, keratinocyte speeds rise upon the disruption of cell adhesions by DECMA-1 [8]. However, the impact on E-cadherin blockade by DECMA-1 on directionality varies significantly with the initial strength of cell-cell adhesion: disruption of E-cadherin junctions enhances the directionality of cells with strong adhesions (high calcium in media), has negligible effects on cells with medium adhesive interactions, and reduces directionality in cells with weaker adhesions (low calcium media). Can we understand why groups of cells might, depending on context, either have an increase or decrease in directionality as adhesion strengths are varied?

**Fig 5:**
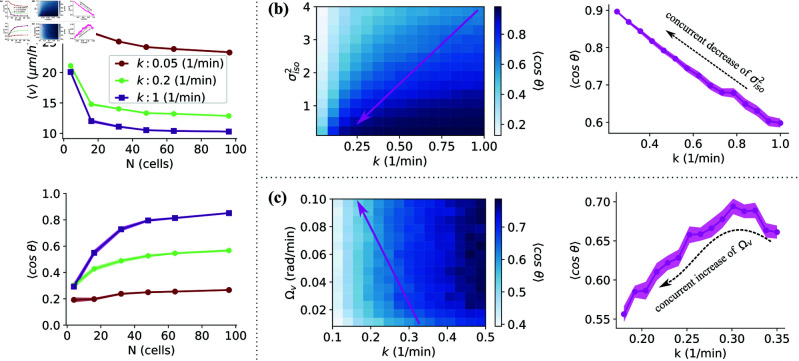
Dependence of directionality on cell-cell adhesion. **(a)** Velocity (top row) and directionality (bottom row) as a function of cell count for different (color coded) spring constants. **(b)** Directionality for different combinations of isotropic variance $\sigma_{\text{iso}}^{2}$ and interaction strength *k* (left). The arrow indicates a linear path of concurrent variations of $\sigma_{\text{iso}}^{2}$ and *k* resulting decreasing directionality shown on the right figure. Here $\Omega_{v}=0.01\;(rad/min)$. **(c)** Directionality for different combinations of alignment rate to velocity $\Omega_{v}$ and interaction strength *k* (left). The arrow indicates a linear path of concurrent variations of $\Omega_{v}$ and *k* resulting decreasing directionality shown on the right figure. Here $\sigma_{\text{iso}}^{2}=2$. The reported values represent averages across 40 simulations with 64 cells $\sigma_{\text{ani}}^{2}=4$. Where applicable the shaded areas represent standard errors of the mean.

With our default parameters, cell speeds decrease and directionalities increase as adhesion strength is increased (Fig 5a). As we saw above, directionality typically increases with the number of cells in the group – though at the weakest adhesion strength, where cells are often separate from the group, cell number only weakly influences directionality. Our cell speed results are consistent with experiment [7, 8], while the directionality results are consistent with those of [7] on MDCK I cells but do not explain the complicated dependence of directionality on adhesion seen in [8]. Why not? One hypothesis is that changing cell-cell adhesion in the experiment regulates multiple factors at once – so that we should model these experimental changes in cell-cell adhesion as changing multiple parameters in our model. For instance, changing cadherin expression is known to regulate cell shape in complex and context-dependent ways [42]. Similarly, changes in cell-cell adhesion may alter cell-substrate interactions [43]. We explore these two hypotheses in our model, to see if it is possible to create decreases in directionality with increasing adhesion strength, or a non-monotonic trend.

We first study what happens if changing E-cadherin strength simultaneously changes cell size or shape, thus altering the precision of cells’ ability to estimate the field direction [16]. As an example for what could happen if cell size changes as E-cadherin strength changes, we show how directionality varies if we vary both $\sigma_{\text{iso}}^{2}$ and *k* in our model in Fig 5b, left. (This is only one possibility; models extending the work of [16] would be necessary to explicitly connect cell size and shape and accuracy.) Increasing the variance of the cells’ estimates naturally leads to reduced directionality across a broad spectrum of adhesion strengths (see Fig 5b, left). As an illustration, suppose that when E-cadherin strength is regulated, this also changes $\sigma_{\text{iso}}^{2}$, following the pink arrow in Fig 5b, left. The directionalities that would be measured as E-cadherin is varied are then shown in Fig 5b, right. If we assume that longer keratinocytes have enhanced overall precision of electric field direction estimate (i.e. reduced $\sigma_{\text{iso}}^{2}$), then noting that keratinocytes become more elongated as adhesions weaken [8], this combination of effects could potentially explain the observed increase in directionality upon disrupting cell-cell adhesions. Since adhesion-dependent shape changes are modest [8], cells still interact with approximately the same neighbors. Thus, we kept the interaction range unchanged in our simulations and focused instead on how accuracy varies with adhesion strength.

A second hypothesis is that cell-cell and cell-substrate interactions may compete [43]. Specifically, formation of cell-cell adhesions, which modulate cellular forces and alignment, can locally downregulate cell-substrate adhesions. Conversely, formation of cell-substrate adhesions, which regulate cell movement through adhesion strength and dynamics, can also weaken cell-cell interactions. In our model, this might mean that cells with weaker cell-cell adhesion are more effective at aligning perpendicular to their own velocity – something we attribute to more effective cell-substrate interactions that could promote reorientation by providing stable anchoring point and enabling efficient signal transduction. We plot the effect of varying $\Omega_{v}$ and *k* separately in Fig 5c, left. For an alternate view of this effect, we can plot directionality as a function of $\Omega_{v}$ (S8a-b Fig). Generally, lower adhesion strength corresponds to reduced directionality across different alignment rates to velocity. However, simultaneous variation of *k* and $\Omega_{v}$ can reverse this trend. We hypothesize that *k* and $\Omega_{v}$ vary simultaneously following the pink arrow in the phase diagram, increasing $\Omega_{v}$ while decreasing *k*. This leads to a weakly non-monotonic behavior, with a slight dip in directionality above k~0.33 ${\text{min}}^{-1}$ (Fig 5c, right). This non-monotonic behavior results from the interplay between the spring constant *k* and $\Omega_{v}$. As *k* decreases, directionality is reduced, while an increase in $\Omega_{v}$ enhances alignment perpendicular to the field, improving the accuracy of individual cells’ electric field direction estimates and leading to higher overall group directionality. The observed dip in directionality, though not dramatic, aligns with experimental findings of a minor dip under similar conditions.

## Discussion

We presented a minimalistic model for collective cellular behavior in the presence of electric fields. We found that, similar to experimental observations, cells that attach to one another can improve their ability to follow electric fields, with increasing accuracy as group size increases. Stronger cell-cell adhesion also resulted in more accurate estimates. We specifically modeled elongated cells and, based on experimental observations and theoretical studies, hypothesized that cells sense the direction of an electric field more effectively when their long axes are orthogonal to the field. Under this assumption, we discovered that groups of cells enhance their field-sensing abilities by combining perpendicular orientation to their time-averaged velocity with nematic alignment with their neighbors. Optimal performance is achieved when there is a balance between these two effects. Specifically, the rate of alignment to time-averaged velocity should be sufficiently high (≥ 0.01 rad/min) to ensure cells tend to align perpendicularly to the field. Simultaneously, the rate of alignment to neighbors should exceed the rate of alignment to velocity for optimal alignment and accuracy. Experiments have previously found inconsistent results on how galvanotactic directionality of cell groups depends on cell-cell adhesion [7, 8]. Our work proposes plausible mechanisms – cell-cell adhesion regulating cell shape or cell-substrate interactions – that could explain why cell-cell adhesion might either increase or decrease directionality depending on the context.

The foundational assumption in our model, inspired by theoretical studies [13, 14, 17], is that cells display significant anisotropy in precision depending on their orientations. To some extent, this is unavoidable – cells with larger length parallel to or perpendicular to the gradient will naturally have a larger “signal” of difference in electric potential or chemical concentration. However, there is no explicit experimental evidence testing this for galvanotaxis that we are aware of – even simpler results like the dependence of directionality on cell size are under-studied [16].

While our study primarily focuses on the response of groups of cells to electric fields that align their long axes perpendicular to the field, in principle, many of our assumptions can be adapted to model other modes of migration, such as chemotaxis, haptotaxis, and durotaxis. One core assumption that would need adjustment is that cells re-estimate the electric field direction only every $\tau_{\text{forget}}$. This relatively long timescale ( ~10 minutes) reflects the time required to transport galvanotactic sensors along the cell surface [15, 16]. (Though this timescale may also be cell-type dependent, as [44] observes much faster reorientation in neutrophil-like HL-60 cells.) In contrast, the best-characterized chemotaxing cell, Dictyostelium, has a much shorter averaging time (a few seconds to tens of seconds) [45–47], and a more detailed model of cell polarization would be necessary to account for these differences. Additionally, application to chemotaxis or to different cell types would require adjustment of other assumptions. Collective chemotaxis of, e.g. neural crest [48] or lymphocytes [49] do not have an obvious tendency to have cells oriented perpendicularly to the chemical gradient – so we expect our considerations here to be less relevant for this case. We also expect that Dictyostelium, which largely travels along its long axis during both chemotaxis and electrotaxis [50, 51], to not be well-described by our model.

Our model does not incorporate mechanisms by which cells’ polarity can influence one another, such as contact inhibition of locomotion (CIL) [52], where cells typically repolarize in the opposite direction upon contact [53]. Including CIL could provide additional pathways through which groups of galvanotaxing cells might enhance their directionality, as has been explored in studies of collective chemotaxis [39, 40, 54]. Furthermore, our representation of physical cell-cell interactions is relatively simplistic, incorporating only a spring-like interaction for adhesion and a phenomenological nematic alignment term, without accounting for cell deformability. While more complex interactions, such as those modeled with extensions of the Gay-Berne potential [55], as applied in [21], could capture these effects, they significantly increase model complexity. Such an approach, while more realistic, would inherently couple interaction strength with intercellular alignment tendencies. In contrast, our simpler model allows us to vary these factors independently.

## Supporting information

S1 Appendix Supplementary Appendix containing additional details on the simulation implementation and further information about the supplementary figures. (PDF)

S1 FigThe average directionality ⟨cosθ⟩ for various combinations of isotropic $\sigma_{\text{iso}}^{2}$ and anisotropic $\sigma_{\text{ani}}^{2}$ variances and alignment rates to velocity.This plot is the same as Fig 1 but at cell-cell the interaction strength of *k* = 1 ${\text{min}}^{-1}$. Isotropic component ($\sigma_{\text{iso}}^{2}$) changes across rows (top to bottom) and anisotropic component ($\sigma_{\text{ani}}^{2}$) across rows (left to right) with specific values shown at right side and bottom of the figure (i.e. Figure (f) show directionalities for $\sigma_{\text{iso}}^{2}=2,\sigma_{\text{ani}}^{2}=4$ ). The averages are over 40 simulations and each simulation is performed with 64 cells. For each simulation the reported directionality is the steady state average over final 5 hours of simulation Fig 1f. Results for different vaues of alignment rates to average velocity are color coded $\Omega_{v}=0$ rad/min (black), $\Omega_{v}=0.01$ rad/min (purple), and $\Omega_{v}=0.1$ rad/min (orange). The averaging time *T* for velocity is set to 1h. The shaded areas represent standard errors, although they may not be easily discernible due to their small size.(TIF)

S2 FigThe average directionality ⟨cosθ⟩ for various combinations of isotropic $\sigma_{\text{iso}}^{2}$ and anisotropic $\sigma_{\text{ani}}^{2}$ variances and alignment rates to velocity.This plot is the same as Fig 1 but with a cell-cell interaction strength of *k* = 0.05 ${\text{min}}^{-1}$. Isotropic component ($\sigma_{\text{iso}}^{2}$) changes across rows (top to bottom) and anisotropic component ($\sigma_{\text{ani}}^{2}$) across rows (left to right) with specific values shown at right side and bottom of the figure (i.e. Figure (f) show directionalities for $\sigma_{\text{iso}}^{2}=2,\sigma_{\text{ani}}^{2}=4$ ). The averages are over 40 simulations and each simulation is performed with 64 cells. For each simulation the reported directionality is the steady state average over final 5 hours of simulation Fig 1f. Results for different vaues of alignment rates to average velocity are color coded $\Omega_{v}=0$ rad/min (black), $\Omega_{v}=0.01$ rad/min (purple), and $\Omega_{v}=0.1$ rad/min (orange). The averaging time *T* for velocity is set to 1h. The shaded areas represent standard errors, although they may not be easily discernible due to their small size.(TIF)

S3 FigCell alignments and directionalities as a function of velocity averaging time.The reported values represent averages across 40 simulations of 64 cells with $\sigma_{\text{iso}}^{2}=2\;\&\;\sigma_{\text{ani}}^{2}=4$ conducted at an interaction strength of **(a)** *k* = 0.05 ${\text{min}}^{-1}$ and **(b)** *k* = 1 ${\text{min}}^{-1}$. In the left column, the absolute value of the cell alignment angle is presented, while the right column displays the corresponding directionality. The shaded areas represent standard errors of the mean.(TIF)

S4 FigWeak adhesion limit – Effect of rates of alignment to velocity ($\Omega_{v}$) and neighbors ($\Omega_{n}$) on directionality (a), alignment (b) and order parameter (c).Each grid value represents an average result over 40 simulations conducted with 64 cells at the interaction strength of k = 0.05 ${\text{min}}^{-1}$ with an averaging time *T* = 1 h with colorbars indicating corresponding numeric values. Example simulation snapshots for alignment rate tuples of **(i)**, $\Omega_{v}=0.001$ rad/min, $\Omega_{n}=0.001$ rad/min; **(ii)**, $\Omega_{v}=0.001$ rad/min, $\Omega_{n}=1$ rad/min; **(iii)**, $\Omega_{v}=0.012$ rad/min, $\Omega_{n}=0.001$ rad/min; **(iv)**, $\Omega_{v}=0.012$ rad/min, $\Omega_{n}=1$ rad/min; **(v)**, $\Omega_{v}=1$ rad/min, $\Omega_{n}=0.001$ rad/min; **(vi)**, $\Omega_{v}=1$ rad/min, $\Omega_{n}=1$ rad/min, also shown in panel **c**. Cells are colored according to their orientation shown on the colorbar. For all simulations $\sigma_{\text{iso}}^{2}=2$ and $\sigma_{\text{ani}}^{2}=4$.(TIF)

S5 FigStrong adhesion limit – Effect of rates of alignment to velocity ($\Omega_{v}$) and neighbors ($\Omega_{n}$) to directionality (a), alignment (b) and order parameter (c).Each grid value represents an average result over 40 simulations conducted with 64 cells at the interaction strength of k = 1 ${\text{min}}^{-1}$ with an averaging time *T* = 1 h with colorbars indicating corresponding numeric values. Example simulation snapshots for alignment rate tuples of **(i)**, $\Omega_{v}=0.001$ rad/min, $\Omega_{n}=0.001$ rad/min; **(ii)**, $\Omega_{v}=0.001$ rad/min, $\Omega_{n}=1$ rad/min; **(iii)**, $\Omega_{v}=0.012$ rad/min, $\Omega_{n}=0.001$ rad/min; **(iv)**, $\Omega_{v}=0.012$ rad/min, $\Omega_{n}=1$ rad/min; **(v)**, $\Omega_{v}=1$ rad/min, $\Omega_{n}=0.001$ rad/min; **(vi)**, $\Omega_{v}=1$ rad/min, $\Omega_{n}=1$ rad/min, also shown in panel **c**. Cells are colored according to their orientation shown on the colorbar. For all simulations $\sigma_{\text{iso}}^{2}=2$ and $\sigma_{\text{ani}}^{2}=4$.(TIF)

S6 FigBehavior of cellular orientation and the direction of the time-averaged velocity $\alpha_{\langle v^{i}\rangle_{T}}$ as alignment rate $\Omega_{v}$ changes for $\Omega_{n}=0.02$ rad/min.Panel **(a)** is the copy of Fig 4a with crosses showing where the exact measurement locations for plots in figures **(b)** and **(c)**. **(b)** Root mean square deviation of cellular orientation ϕ from the direction of orthogonal to time averaged velocity $\alpha_{\langle v^{i}\rangle_{T}}\;+\;\pi /2$ for different alignment rates $\Omega_{v}$. **(b)** Root mean square deviation of the direction of orthogonal to time averaged velocity $\alpha_{\langle v^{i}\rangle_{T}}\;+\;\pi /2$ from favorable vertical π/2 orientation for different alignment rates $\Omega_{v}$. Each value in panels **(b)** and **(c)** represent an average over 40 simulations conducted with 64 cells that have isotropic and anisotropic variance components of $\sigma_{\text{iso}}^{2}=2$ & $\sigma_{\text{ani}}^{2}=4$ at the interaction strength of k = 0.2 ${\text{min}}^{-1}$ with an averaging time *T* = 1 h. Error bars indicate standard errors.(TIF)

S7 FigEffect of rates of alignment to velocity ($\Omega_{v}$) and neighbors ($\Omega_{n}$) to directionality (a)&(d), alignment (b)&(e) and order parameter (c)&(f).Phase diagrams on the top row show simulations for cells with isotropic and anisotropic components of variance of $\sigma_{\text{iso}}^{2}=2$ & $\sigma_{\text{ani}}^{2}=6$, and bottom row represents simulations for $\sigma_{\text{iso}}^{2}=0.5$ & $\sigma_{\text{ani}}^{2}=4$ Each grid value represents an average result over 40 simulations conducted with 64 cells at the interaction strength of k = 0.2 ${\text{min}}^{-1}$ with an averaging time *T* = 1 h with colorbars indicating corresponding numeric values.(TIF)

S8 FigDependence of directionality on alignment rate to velocity $\Omega_{v}$ for selected cell-cell adhesion strengths.**(a)** Replication of the Fig 5c, left with arrows showing the spring constants used while varying $\Omega_{v}$ for the plot on panel **(b)**. Colors of the arrows correspond to the colors on the panel **(b)**. **(b)** Directionality as a function of alignment rate to velocity for different values of interaction strength *k*. Even though decreasing adhesion strength generally results lower directionalities, the dotted cyan arrow shows a possible path of increase in cell orientation rate as adhesion decreases that could lead to higher directionality. The reported values represent averages across 40 simulations with 64 cells with $\sigma_{\text{iso}}^{2}=2$ and $\sigma_{\text{ani}}^{2}=4$. The shaded areas represent standard errors of the mean.(TIF)

S9 FigCell orientation histograms for various combinations of interaction strengths and diffusion coefficients.Interaction strength varies across rows (top to bottom) and diffusion coefficient across columns (left to right) with specific values of spring constant shown at right side and diffusion coefficient at bottom of the figure (i.e. panel **(e)** shows orientations for $k=0.2\;{\text{min}}^{-1}$ and $D_{r}=0.003\;rad^{2}/min$ ). Each histogram is compiled from the final snapshot data of 40 simulations, each featuring 64 cells. Cells do not align to neighbors $\Omega_{n}=0\text{ rad/min}$, align to average velocity (averaging time *T* = 1 h) at the rate of $\Omega_{v}=0.01\;rad/min$, and have $\sigma_{\text{iso}}^{2}=2$ and $\sigma_{\text{ani}}^{2}=4$.(TIF)

S1 MovieExample simulation video of cells with $\sigma_{\text{iso}}^{2}=2$ and $\sigma_{\text{ani}}^{2}=4$ at the interaction strength of k = 0.2 ${\text{min}}^{-1}$.(MP4)

S2 MovieExample simulation video of cells with $\sigma_{\text{iso}}^{2}=2$ and $\sigma_{\text{ani}}^{2}=4$ at the interaction strength of k = 1 ${\text{min}}^{-1}$.(MP4)

S3 MovieExample simulation video of cells with $\sigma_{\text{iso}}^{2}=2$ and $\sigma_{\text{ani}}^{2}=4$ at the interaction strength of k = 0.05 ${\text{min}}^{-1}$.(MP4)
