# Supplementary material for: Does nematic order allow groups of elongated cells to sense electric fields better?
Source: PLoS One. 2025 Jun 25;20(6):e0325800. doi: 10.1371/journal.pone.0325800 (PMC12193095; doi:10.1371/journal.pone.0325800)
Supplement: S1 Appendix — (PDF) [file pone.0325800.s001.pdf]

Supplementary Information Text For: Does nematic order allow groups of elongated cells to sense electric fields better?

Kurmanbek Kaiyrbekov, Brian A. Camley

Details of numerical methods

Initialization of simulations

We conduct $\mathcal{S} = 40$ simulations for each parameter configuration, with simulations indexed from 1 to 40. To enhance comparability and reproducibility, we initialize the random seed to its corresponding simulation index at the beginning of each simulation. Cells are initially placed at random coordinates, maintaining a minimum distance of 25 μm from each other, with a density of $\rho_i = 1024$ cells/mm^2 near the center of the simulation box. This setup guarantees no isolated cells and fosters the formation of a single cell cluster at the start. The initial orientations of the cells are randomly assigned, following a uniform distribution $\mathcal{U}\,[0, 2\pi]$.

Numerical integration

In order to monitor a cell's neighboring cells, we maintain a list containing all cells within a distance r_{nn} of the given cell. This list is regularly updated each time any cell moves a distance of Δd_{nn} since the previous update. It's important to note that a cell doesn't interact with all the cells on the neighbor list, but only with those within the defined cutoff distance r_c.

At the beginning of each iteration, we calculate the interaction forces and torques acting on every cell. Subsequently, we employ numerical integration to solve the equations of motion as follows: For each cell i, the positions are updated every simulation step as:

$$\mathbf{r}^i(t + \Delta t) = \mathbf{r}^i(t) + \left(\mathbf{p}^i + \sum_{j \underset{n}{\sim} i} \mathbf{F}^{ij} \right) \Delta t \tag{S1}$$

where as before the sum is over the cells that are within the interaction cutoff distance r_c. Cells synchronously update their polarity at intervals of τ_{forget}, keeping this updated polarity constant for the subsequent period of τ_{forget}. Cell orientations are updated via Euler-Maruyama method [1]:

$$\begin{aligned} \phi^i(t + \Delta t) = \quad & \phi^i(t) \\ & - \Omega_v \sin(2[\phi^i - (\alpha_{\langle v^i \rangle_T} + \pi/2)])\Delta t \\ & - \Omega_n \sum_{j \underset{n}{\sim} i} \sin(2[\phi^i - \phi^j])\Delta t \\ & + \sqrt{2D_r}\Gamma \end{aligned} \tag{S2}$$

where the summation is over neighbor cells. In each iteration, cells update $\alpha_{\langle v^i \rangle_T}$ to retain a moving average of the angle. The term $\Gamma = \int_t^{t+\Delta t} \xi_r(t)dt$, hence $\langle \Gamma \rangle = 0$ and $\langle \Gamma^2 \rangle = \Delta t$. Numerically, every simulation timestep we sample Γ from normal distribution $\mathcal{N}(\mu = 0, \sigma^2 = \Delta t)$ i.e. $\Gamma \sim \mathcal{N}(\mu = 0, \sigma^2 = \Delta t)$.

Table S1. Simulation parameters

Parameter	Meaning	Value
r_c	Interaction cutoff radius	50 μm
r_{eq}	Equilibrium length of spring	40 μm
r_{nn}	Cutoff radius of neighbor lists	60 μm
Δd_{nn}	Threshold displacement to initiate neighbor list update	8 μm
D_r	Rotational diffusion coefficient	0.003 rad^2/min
τ_{forget}	Cell repolarization timescale (period)	10 min
Δt	Simulation time step	0.001 min

Parameter setting

We think of our cells as ellipses having a long axis radius of $a = 20\ \mu m$ and short axis radius of $b = 10\ \mu m$. However, a and b do not actually affect the simulation except for setting the interaction cutoff distance, only the plotting. Within the simulation, the cell is only characterized by its long axis orientation ϕ, and the cell-cell interactions are simple isotropic springs, which set the cell-cell distances. We set the interaction cutoff distance (r_c), spring equilibrium length (r_{eq}), and neighbor list tracking radius (r_{nn}) were set to ensure most interactions are only with reasonable neighboring cells, given the cell size we defined. Note that the interaction cutoff distance (r_c) is the maximum distance within which cells interact. The neighbor tracking distance (r_{nn}), on the other hand, is a larger distance (i.e. $r_{nn} > r_c$) used to maintain a list of nearby cells, to ensure efficient force calculations and avoid searches for nearby cells at every simulation step. To ensure that neighbor list is accurate it is updated if any of the cells in simulation moves by more than a distance Δd_{nn} since the last update.

The rotational diffusion coefficient was calibrated to ensure cells exhibited behavior consistent with typical observations. We performed simulations for various D_r values and interaction strengths using our default choice of variances. Consistent with expectations cells were more aligned for smaller diffusion coefficient (Fig S9a,d,g) and disordered for large D_r (Fig S9c,f,i). We select a value of 0.003 rad^2/min (see Fig S9b,e,f) since its orientation distributions for medium interaction strength $k = 0.2$ min^{-1} roughly resembled experimental histograms [2]. The complete set of parameters is detailed in S1 Table. Parameters not listed in this table were varied for the purposes of this study, and their specific values are provided in the main text.

Center of mass motion

The center of mass (c.o.m.) of a cell cluster evolves via simple equation of motion, which can be computed by taking sum of Eqn. (1) over cells:

$$\frac{1}{N}\sum_{i=1}^{N}\frac{\partial \mathbf{r^i}}{\partial t} = \frac{1}{N}\sum_{i=1}^{N}\mathbf{p^i} + \frac{1}{N}\sum_{i=1}^{N}\sum_{j}\mathbf{F}^{ij} \tag{S3}$$

Owing to Newton's third law, the last term cancels out, i.e., $\sum_{i=1}^{N}\sum_{j}\mathbf{F}^{ij} = 0$. Incorporating summation inside the partial derivative of Eqn. (S3) and defining the coordinate of the center of mass as $\mathbf{r}^{c.o.m} = \sum_{i=1}^{N}\mathbf{r}^i/N$ we reach:

$$\frac{\partial \mathbf{r}^{c.o.m}}{\partial t} = \frac{1}{N}\sum_{i=1}^{N}\mathbf{p}^i \tag{S4}$$

Since a cell i polarizes towards its estimate of field direction (i.e. $\mathbf{p}^i = \cos(\zeta^i)\hat{x} + \sin(\zeta^i)\hat{y}$, where $\zeta^i \sim VM(0, \kappa^i)$), the center of mass migrates towards

the group's mean estimated direction. Due to the law of large numbers, the accuracy of average estimate improves for larger N, hence the c.o.m. of larger groups of cells follow electric field more efficiently.

The migration of the c.o.m. is independent of the adhesion strength k; however, k influences the extent to which individual cells adhere to the c.o.m. within the displacement measurement interval of 30 minutes. A higher k ensures that cells robustly track the c.o.m., whereas a lower k allows individual cells more freedom to deviate from the group's migration path.

Directionalities for different combinations of isotropic and anisotropic variance for weakly and strongly interacting cells

In the main text, we present directionalities for various combinations of isotropic and anisotropic components of variance at a default cell-cell interaction strength of $k = 0.2\ \text{min}^{-1}$. When the adhesion strength is increased to $k = 1\ \text{min}^{-1}$, the trends remain similar across all components of anisotropic and isotropic variance, with directionalities improving with cluster size. In this case, directionalities are consistently higher since cells more effectively follow the center of mass (see Fig S1). Sufficient anisotropy in the components is necessary to observe the benefits of alignment orthogonally to velocity in this scenario. For weaker cellular interactions ($k = 0.05\ \text{min}^{-1}$), directionalities are lower, and there is little dependence on cluster size since cells behave more like individual elements (see Fig S2). The benefits of alignment are observed only when cells are precise enough to accurately estimate the field direction (i.e. $\sigma^2_{\text{iso}} = 0.5$, Fig S2a-c).

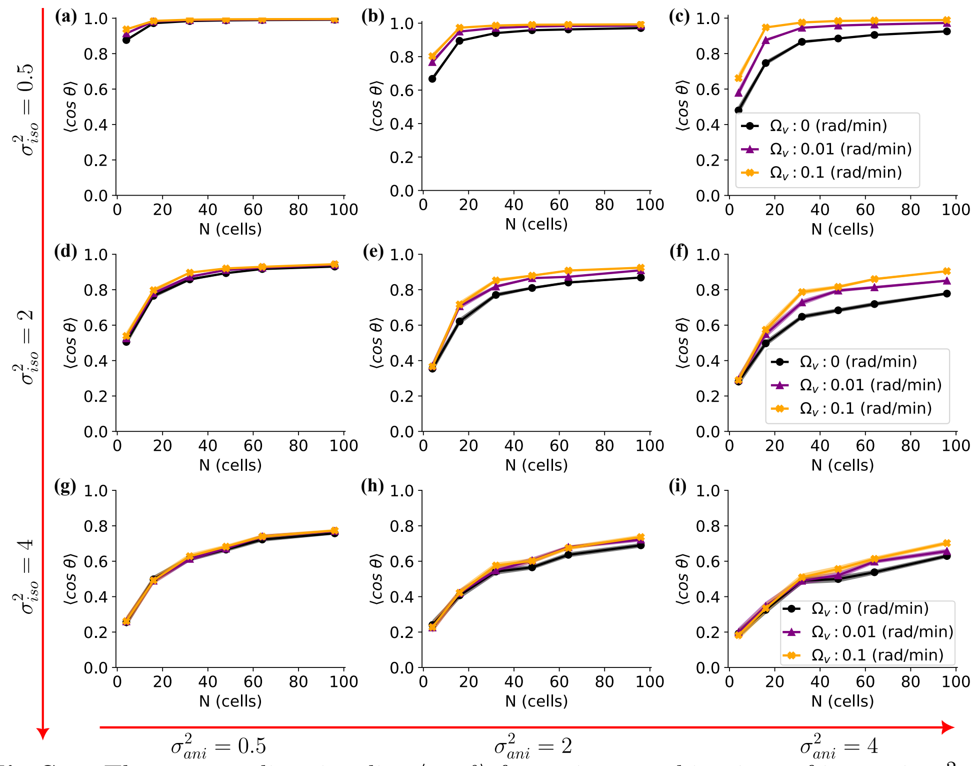

Fig S1. The average directionality $\langle \cos\theta \rangle$ for various combinations of isotropic σ^2_{iso} and anisotropic σ^2_{ani} variances and alignment rates to velocity. This plot is the same as Fig 1 but at cell-cell the interaction strength of $k = 1$ min^{-1}. Isotropic component (σ^2_{iso}) changes across rows (top to bottom) and anisotropic component (σ^2_{ani}) across rows (left to right) with specific values shown at right side and bottom of the figure (i.e. Figure (f) show directionalities for $\sigma^2_{\text{iso}} = 2, \sigma^2_{\text{ani}} = 4$). The averages are over 40 simulations and each simulation is performed with 64 cells. For each simulation the reported directionality is the steady state average over final 5 hours of simulation Fig 1f. Results for different vaues of alignment rates to average velocity are color coded $\Omega_v = 0$ rad/min (black), $\Omega_v = 0.01$ rad/min (purple), and $\Omega_v = 0.1$ rad/min (orange). The averaging time T for velocity is set to 1h. The shaded areas represent standard errors, although they may not be easily discernible due to their small size.

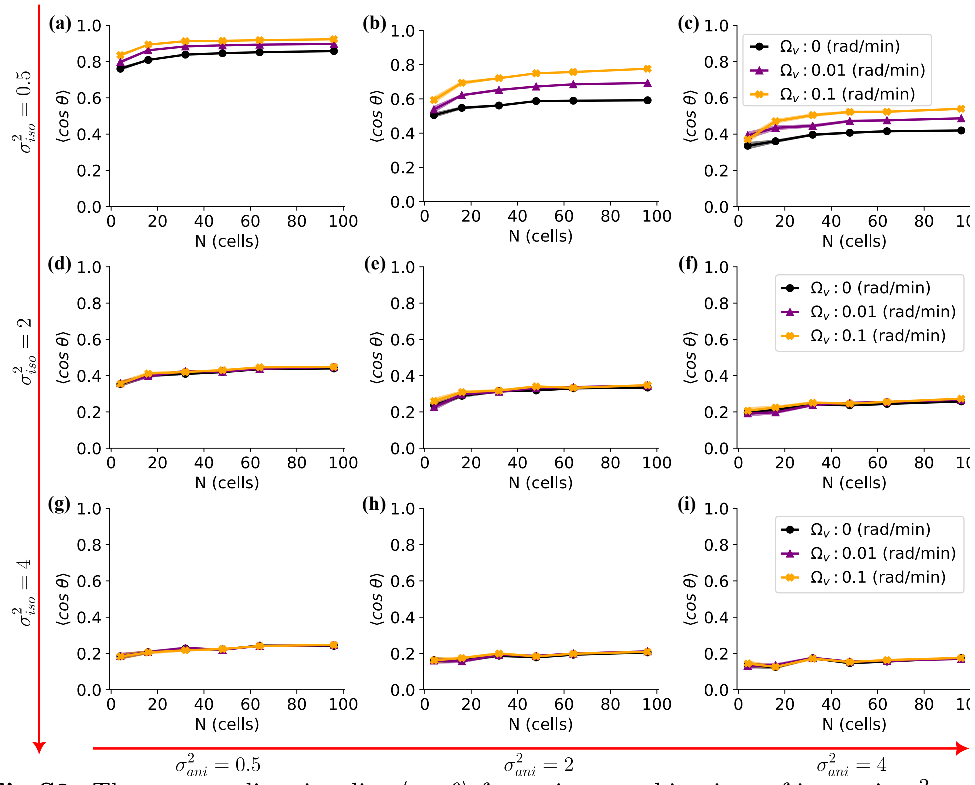

Fig S2. The average directionality $\langle \cos\theta \rangle$ for various combinations of isotropic σ^2_{iso} and anisotropic σ^2_{ani} variances and alignment rates to velocity. This plot is the same as Fig 1 but with a cell-cell interaction strength of $k = 0.05$ min^{-1}. Isotropic component (σ^2_{iso}) changes across rows (top to bottom) and anisotropic component (σ^2_{ani}) across rows (left to right) with specific values shown at right side and bottom of the figure (i.e. Figure (f) show directionalities for $\sigma^2_{\text{iso}} = 2, \sigma^2_{\text{ani}} = 4$). The averages are over 40 simulations and each simulation is performed with 64 cells. For each simulation the reported directionality is the steady state average over final 5 hours of simulation Fig 1f. Results for different vaues of alignment rates to average velocity are color coded $\Omega_v = 0$ rad/min (black), $\Omega_v = 0.01$ rad/min (purple), and $\Omega_v = 0.1$ rad/min (orange). The averaging time T for velocity is set to 1h. The shaded areas represent standard errors, although they may not be easily discernible due to their small size.

Velocity averaging period for weakly and strongly interacting cells

Varying the averaging period T for computing the direction of time-averaged velocity $\alpha_{\langle \boldsymbol{v}^i \rangle_T}$ will generally allow cells to improve their estimates. However, the effects may be weak, especially if cell-cell interaction is weak. We know that the velocity of an individual cell in the limit of weakly interacting cells ($k = 0.05$ min^{-1}) is a very noisy estimator of the direction of the electric field, because cell velocities do not effectively follow the center of mass. In this limit, while increasing the averaging time slightly improves vertical alignment for higher Ω_v, this is not a large enough effect to significantly enhance cluster directionality (Fig S3a,left-right). If we averaged over much much larger values of T, we would expect the trend shown in the orientation in Fig S3a to continue, and this to eventually lead to effects on the cell directionality. By contrast, changing the averaging time at $k = 1$ min^{-1} has a similar effect as at the default interaction strength of $k = 0.2$ min^{-1}. Specifically, as T is increased for faster alignment rates Ω_v, cells become more vertically aligned, and their directionality increases (see Fig S3b,left-right).

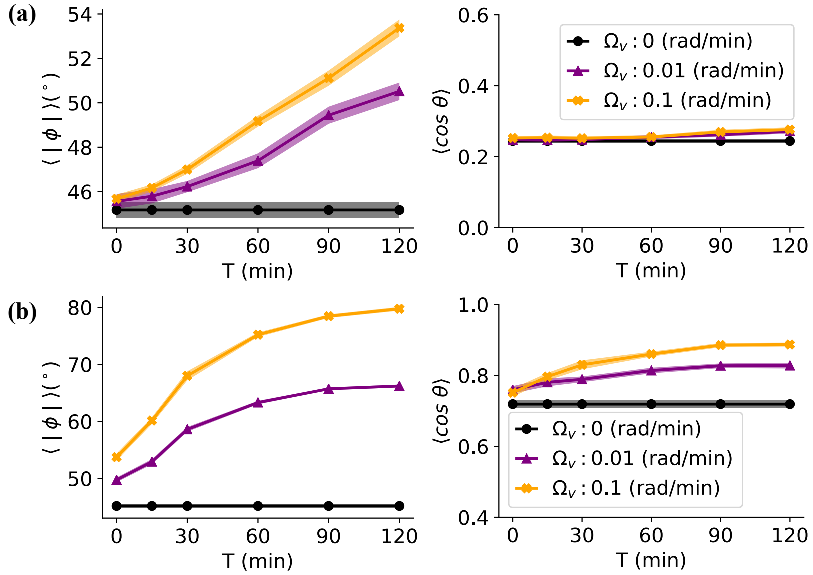

Fig S3. Cell alignments and directionalities as a function of velocity averaging time. The reported values represent averages across 40 simulations of 64 cells with $\sigma^2_{\text{iso}} = 2$ & $\sigma^2_{\text{ani}} = 4$ conducted at an interaction strength of **(a)** $k = 0.05$ min^{-1} and **(b)** $k = 1$ min^{-1}. In the left column, the absolute value of the cell alignment angle is presented, while the right column displays the corresponding directionality. The shaded areas represent standard errors of the mean.

Interplay of alignment to velocity and to neighbors for weakly and strongly interacting cells

Weakly interacting ($k = 0.05$min^{-1}) cells exhibit lower directionalities, poorer alignment, and reduced order across a wide range of alignment rates (Ω_v and Ω_n) compared to our default settings (refer to Fig 4a-c, S4). However, they still achieve high tissue order at large alignment rates to neighbors since most cells are in proximity and

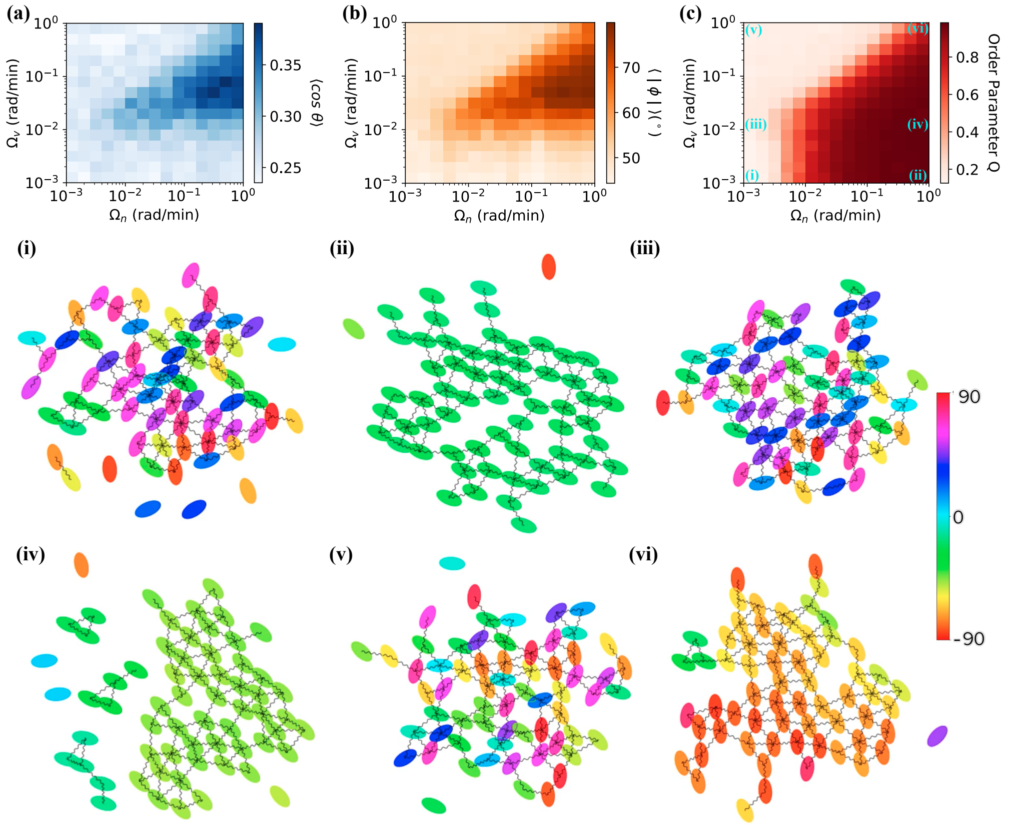

Fig S4. Weak adhesion limit – Effect of rates of alignment to velocity (Ω_v) and neighbors (Ω_n) on directionality **(a)**, alignment **(b)** and order parameter **(c)**. Each grid value represents an average result over 40 simulations conducted with 64 cells at the interaction strength of k = 0.05 min^{-1} with an averaging time T = 1 h with colorbars indicating corresponding numeric values. Example simulation snapshots for alignment rate tuples of **(i)**, $\Omega_v = 0.001$ rad/min, $\Omega_n = 0.001$ rad/min; **(ii)**, $\Omega_v = 0.001$ rad/min, $\Omega_n = 1$ rad/min; **(iii)**, $\Omega_v = 0.012$ rad/min, $\Omega_n = 0.001$ rad/min; **(iv)**, $\Omega_v = 0.012$ rad/min, $\Omega_n = 1$ rad/min; **(v)**, $\Omega_v = 1$ rad/min, $\Omega_n = 0.001$ rad/min; **(vi)**, $\Omega_v = 1$ rad/min, $\Omega_n = 1$ rad/min, also shown in panel **c**. Cells are colored according to their orientation shown on the colorbar. For all simulations $\sigma^2_{\text{iso}} = 2$ and $\sigma^2_{\text{ani}} = 4$.

tend to align with each other. Additionally, for sufficient alignment orthogonally to velocity ($\Omega_v \geq 0.01$ rad/min), faster alignment to neighbors causes cells to assume more vertical orientations and achieve higher directionalities.

Strongly interacting ($k = 1\text{min}^{-1}$) cells demonstrate high directionality, more vertical alignment, and greater order for $\Omega_v \geq 0.01$ rad/min regardless of the alignment rate to neighbors (see to Fig S5). This is because these cells effectively follow the center of mass of the cluster, making $\alpha_{\langle \boldsymbol{v}^i \rangle_T}$ a much more reliable estimate of the field direction. This reliability allows cells to assume more favorable orientations at higher alignment rates to velocity. Nonetheless, we still observe that directionalities and alignment generally improve as the alignment rates to neighbors increase for a constant Ω_v

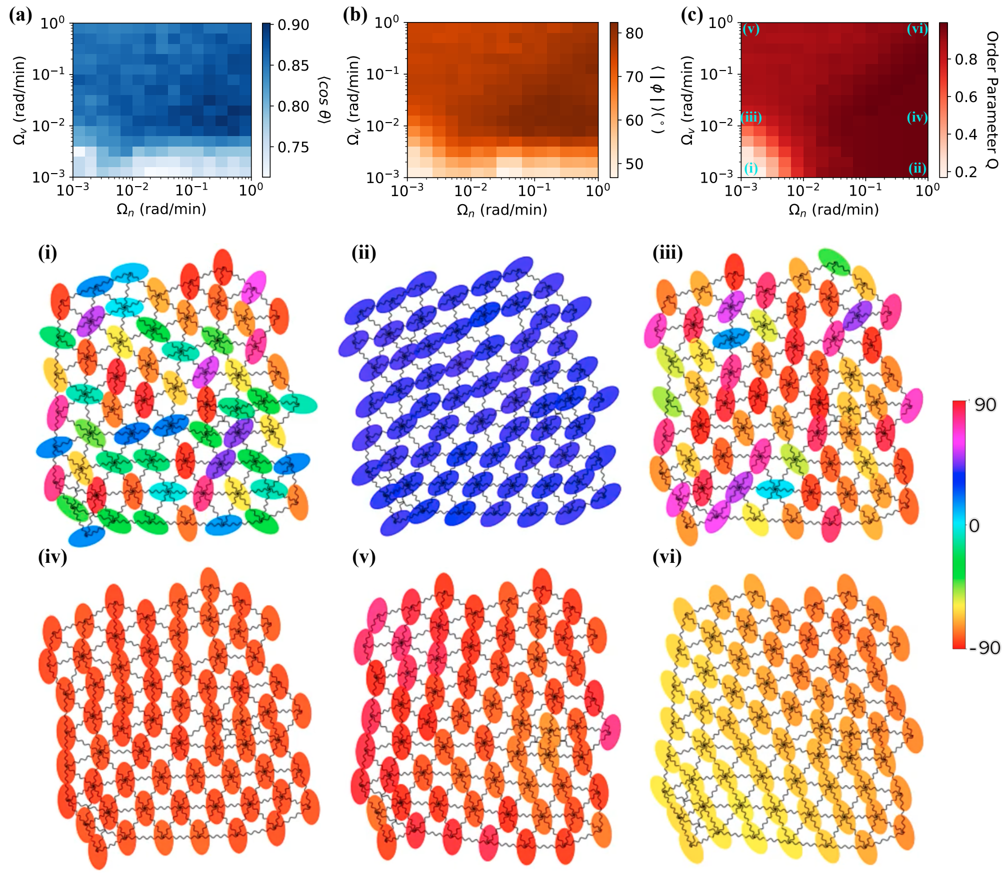

Fig S5. Strong adhesion limit – Effect of rates of alignment to velocity (Ω_v) and neighbors (Ω_n) to directionality **(a)**, alignment **(b)** and order parameter **(c)**. Each grid value represents an average result over 40 simulations conducted with 64 cells at the interaction strength of k = 1 min^{-1} with an averaging time $T = 1$ h with colorbars indicating corresponding numeric values. Example simulation snapshots for alignment rate tuples of **(i)**, $\Omega_v = 0.001$ rad/min, $\Omega_n = 0.001$ rad/min; **(ii)**, $\Omega_v = 0.001$ rad/min, $\Omega_n = 1$ rad/min; **(iii)**, $\Omega_v = 0.012$ rad/min, $\Omega_n = 0.001$ rad/min; **(iv)**, $\Omega_v = 0.012$ rad/min, $\Omega_n = 1$ rad/min; **(v)**, $\Omega_v = 1$ rad/min, $\Omega_n = 0.001$ rad/min; **(vi)**, $\Omega_v = 1$ rad/min, $\Omega_n = 1$ rad/min, also shown in panel **c**. Cells are colored according to their orientation shown on the colorbar. For all simulations $\sigma^2_{\text{iso}} = 2$ and $\sigma^2_{\text{ani}} = 4$.

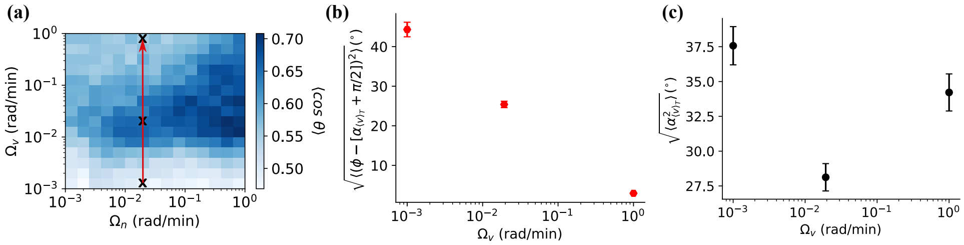

Fig S6. Behavior of cellular orientation and the direction of the time-averaged velocity $\alpha_{\langle \boldsymbol{v}^i \rangle_T}$ as alignment rate Ω_v changes for $\Omega_n = 0.02$ rad/min. Panel **(a)** is the copy of Fig 4a with crosses showing where the exact measurement locations for plots in figures **(b)** and **(c)**. **(b)** Root mean square deviation of cellular orientation ϕ from the direction of orthogonal to time averaged velocity $\alpha_{\langle \boldsymbol{v}^i \rangle_T} + \pi/2$ for different alignment rates Ω_v. **(b)** Root mean square deviation of the direction of orthogonal to time averaged velocity $\alpha_{\langle \boldsymbol{v}^i \rangle_T} + \pi/2$ from favorable vertical $\pi/2$ orientation for different alignment rates Ω_v. Each value in panels **(b)** and **(c)** represent an average over 40 simulations conducted with 64 cells that have isotropic and anisotropic variance components of $\sigma^2_{\text{iso}} = 2$ & $\sigma^2_{\text{ani}} = 4$ at the interaction strength of k = 0.2 min^{-1} with an averaging time $T = 1$ h. Error bars indicate standard errors.

Exploring non-monotonic behavior in directionality phase diagram

For fixed alignment rate to neighbors $\Omega_n \sim 0.02$ rad/min we can see that at low Ω_v cells have low directionality, then directionality improves as we increase alignment rate to velocity (see Fig S6a). However, at excessively high Ω_v values, directionality decreases again leading to non-monotonic behavior. This happens due to two complementary reasons:

1. As we increase Ω_v cells rapidly align their long axis perpendicular to their own averaged velocity, neglecting neighbor information (see Fig S6b).

2. The angle perpendicular to time-averaged velocity $\alpha_{\langle \boldsymbol{v}^i \rangle_T} + \pi/2$ estimates are noisy approximations of the favorable angle perpendicular to the electric field ($\pi/2$) (see Fig S6c).

Thus, in order to maintain most accurate field direction estimates cells need intermediate $\Omega_v \sim \Omega_n$ values incorporating neighbor information that improves vertical alignment of cells and directionality.

Increasing anisotropy by raising σ^2_{ani} to 6 while keeping $\sigma^2_{\text{iso}} = 2$ does not alter the patterns in directionality, alignment, and order (Fig S7a-c). In this regime, cells become less accurate due to increase in σ^2_{ani}. Hence, the cells' estimate of the electric field direction and subsequently the estimate of the favorable vertical orientation (i.e., $\alpha_{\langle \boldsymbol{v}^i \rangle_T} + \pi/2$) do not become more reliable, leading to the same non-monotonic behavior observed with the default variance components (i.e. $\sigma^2_{\text{iso}} = 2$ & $\sigma^2_{\text{ani}} = 4$). Conversely, maintaining $\sigma^2_{\text{ani}} = 4$ and decreasing σ^2_{iso} to 0.5 significantly improves the accuracy of cells in any orientation, making $\alpha_{\langle \boldsymbol{v}^i \rangle_T} + \pi/2$ a more reliable estimate of the favorable vertical orientation (i.e. $\pi/2$). This enhancement significantly diminishes non-monotonic behavior and leads to higher directionality, order, and favorable alignment, provided the cells tend to align orthogonally to the time-averaged velocity (see Fig S7d-f). Therefore, for non-monotonic behavior to occur, the cells need a sufficiently large combination of σ^2_{iso} and σ^2_{ani} values, that make their estimates of the field direction noisy.

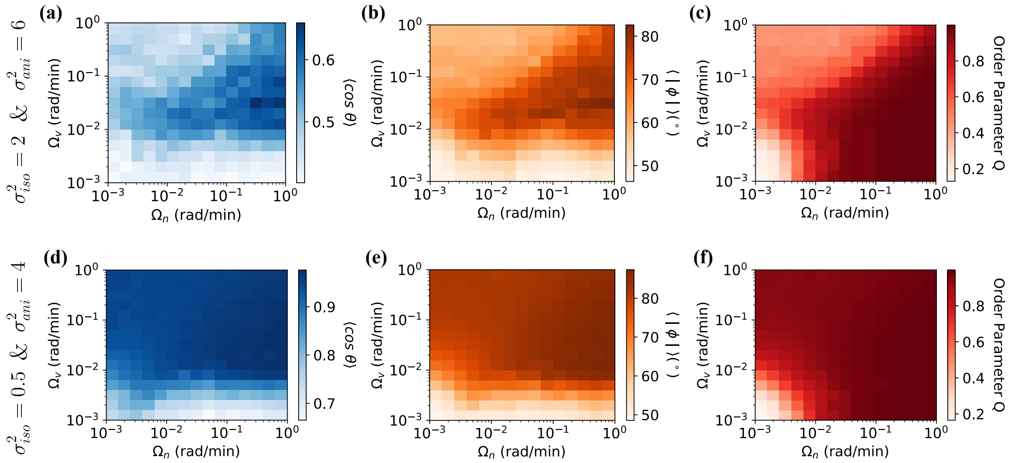

Fig S7. Effect of rates of alignment to velocity (Ω_v) and neighbors (Ω_n) to directionality **(a)&(d)**, alignment **(b)&(e)** and order parameter **(c)&(f)**. Phase diagrams on the top row show simulations for cells with isotropic and anisotropic components of variance of $\sigma^2_{\text{iso}} = 2$ & $\sigma^2_{\text{ani}} = 6$, and bottom row represents simulations for $\sigma^2_{\text{iso}} = 0.5$ & $\sigma^2_{\text{ani}} = 4$ Each grid value represents an average result over 40 simulations conducted with 64 cells at the interaction strength of k = 0.2 min^{-1} with an averaging time $T = 1$ h with colorbars indicating corresponding numeric values.

Reversing adhesion strength dependent behavior by covarying alignment rate to velocity

Adhesion strength is directly proportional to directionality; lower adhesion strength deteriorates directionality (Fig 5a, bottom). This relationship holds true across a wide range of alignment rates to time-averaged velocity (see Fig S8a-b). However, if we simultaneously increase the alignment rate to velocity while decreasing adhesion strength – jumping from the red curve to the black curve as indicated by the cyan dotted arrow – the trend can reverse, resulting in higher directionality despite lower cell-cell adhesion. Therefore, if cells increase their alignment rate to time-averaged velocity as cell-cell interaction strength decreases, they can improve directionality.

Effect of rotational diffusion coefficient on alignment of cells

The rotational diffusion coefficient characterizes the random reorientation of the cell's long axis. To select an appropriate rotational diffusion coefficient that aligns with experimental observations, we performed simulations with various D_r values for different cell-cell adhesion strengths. For all adhesion strengths, we observed that increasing D_r made cell orientations more isotropic (Fig S9a-i). This is expected since a higher D_r indicates quicker randomization of a cell's orientation over time. We selected $D_r = 0.003$ rad^2/min because it best qualitatively matched the experimental distribution of orientations observed in [2]. We set alignment rate with neighbors $\Omega_n = 0$ rad/min in our simulations for selecting the diffusion coefficient. We chose to ignore the effect of intercellular alignment because the experimental histograms were created at the approximate confluence of 70% and we didn't notice significant alignment of cells with neighbors prior to application of the electric field in the relevant supplementary video in [2].

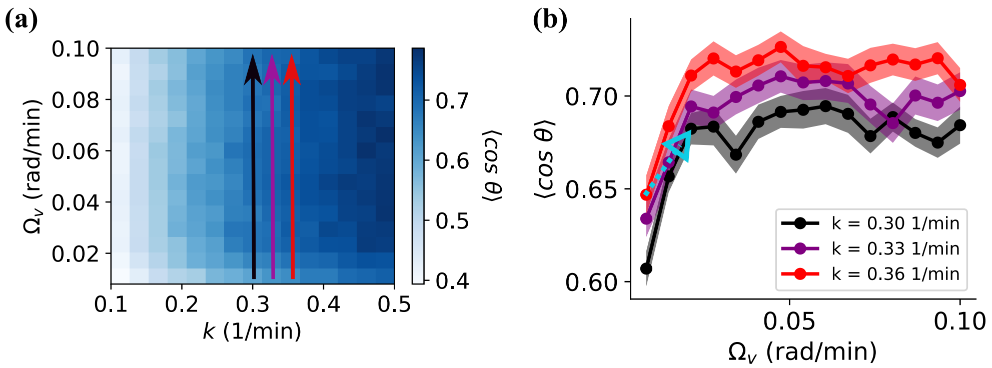

Fig S8. Dependence of directionality on alignment rate to velocity Ω_v for selected cell-cell adhesion strengths. **(a)** Replication of the Fig 5c, left with arrows showing the spring constants used while varying Ω_v for the plot on panel **(b)**. Colors of the arrows correspond to the colors on the panel **(b)**. **(b)** Directionality as a function of alignment rate to velocity for different values of interaction strength k. Even though decreasing adhesion strength generally results lower directionalities, the dotted cyan arrow shows a possible path of increase in cell orientation rate as adhesion decreases that could lead to higher directionality. The reported values represent averages across 40 simulations with 64 cells with $\sigma^2_{\text{iso}} = 2$ and $\sigma^2_{\text{ani}} = 4$. The shaded areas represent standard errors of the mean.

References

1. Kloeden PE, Platen E, Kloeden PE, Platen E. Stochastic differential equations. Springer; 1992.

2. Zajdel TJ, Shim G, Wang L, Rossello-Martinez A, Cohen DJ. SCHEEPDOG: programming electric cues to dynamically herd large-scale cell migration. Cell systems. 2020;10(6):506–514.

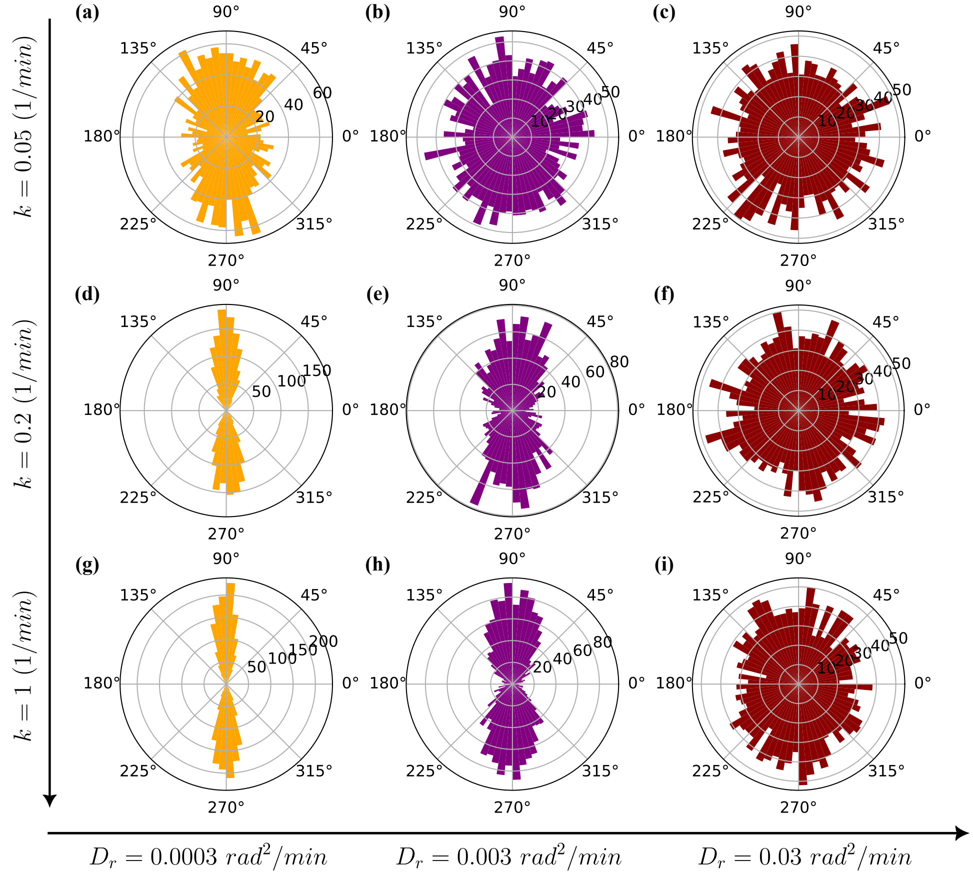

Fig S9. Cell orientation histograms for various combinations of interaction strengths and diffusion coefficients. Interaction strength varies across rows (top to bottom) and diffusion coefficient across columns (left to right) with specific values of spring constant shown at right side and diffusion coefficient at bottom of the figure (i.e. panel **(e)** shows orientations for $k = 0.2$ min^{-1} and $D_r = 0.003\ rad^2/min$). Each histogram is compiled from the final snapshot data of 40 simulations, each featuring 64 cells. Cells do not align to neighbors $\Omega_n = 0$ rad/min, align to average velocity (averaging time T = 1 h) at the rate of $\Omega_v = 0.01\ rad/min$, and have $\sigma^2_{\text{iso}} = 2$ and $\sigma^2_{\text{ani}} = 4$.
