# Supplementary figures and images for: Does nematic order allow groups of elongated cells to sense electric fields better?
Source: PLoS One. 2025 Jun 25;20(6):e0325800. doi: 10.1371/journal.pone.0325800 (PMC12193095; doi:10.1371/journal.pone.0325800)

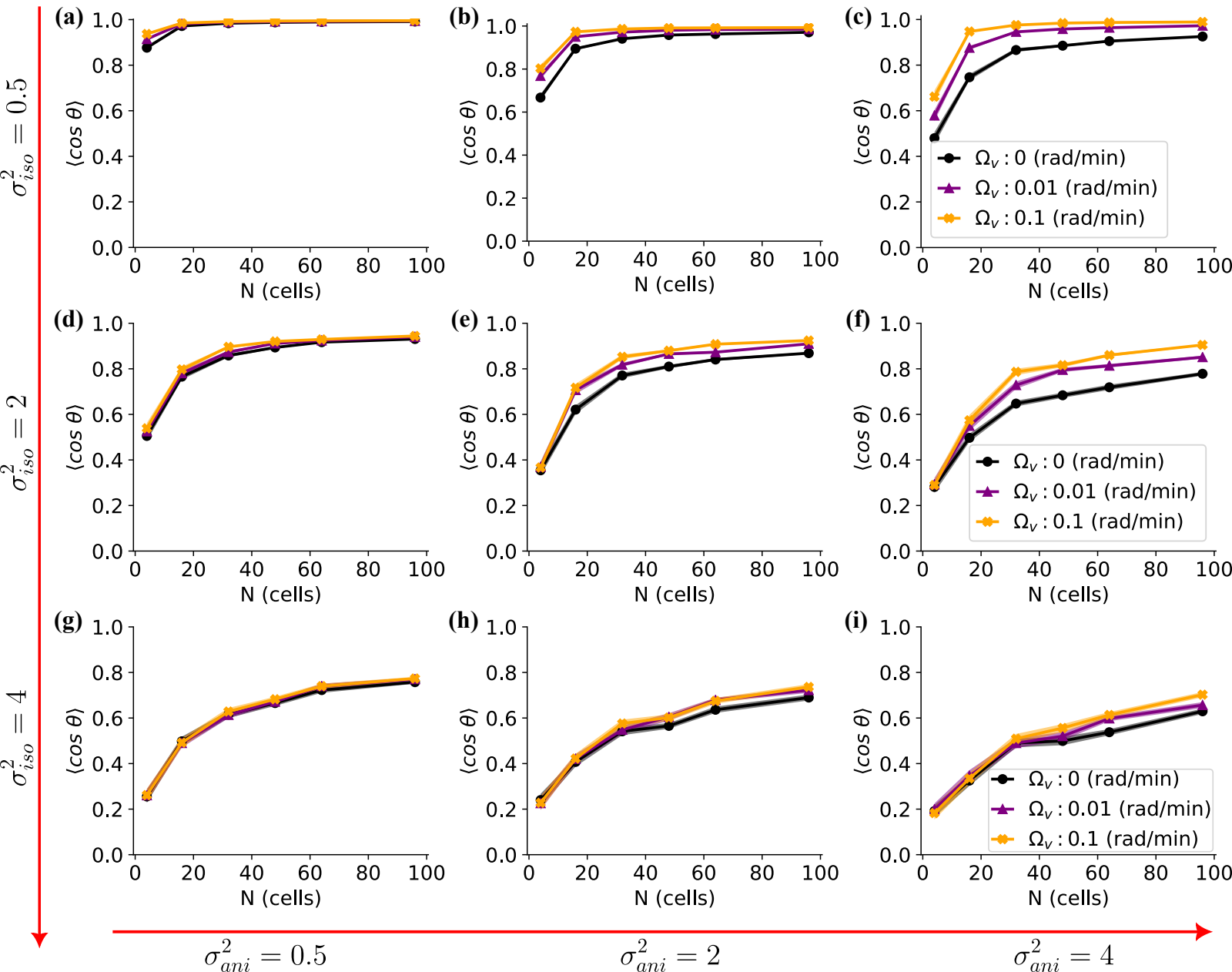

Supplement: S1 Fig — This plot is the same as Fig 1 but at cell-cell the interaction strength of k = 1 min−1. Isotropic component (σiso2) changes across rows (top to bottom) and anisotropic component (σani2) across rows (left to right) with specific values shown at right side and bottom of the figure (i.e. Figure (f) show directionalities for σiso2=2,σani2=4 ). The averages are over 40 simulations and each simulation is performed with 64 cells. For each simulation the reported directionality is the steady state average over final 5 hours of simulation Fig 1f. Results for different vaues of alignment rates to average velocity are color coded Ωv=0 rad/min (black), Ωv=0.01 rad/min (purple), and Ωv=0.1 rad/min (orange). The averaging time T for velocity is set to 1h. The shaded areas represent standard errors, although they may not be easily discernible due to their small size. (TIF) [file pone.0325800.s002.pdf]

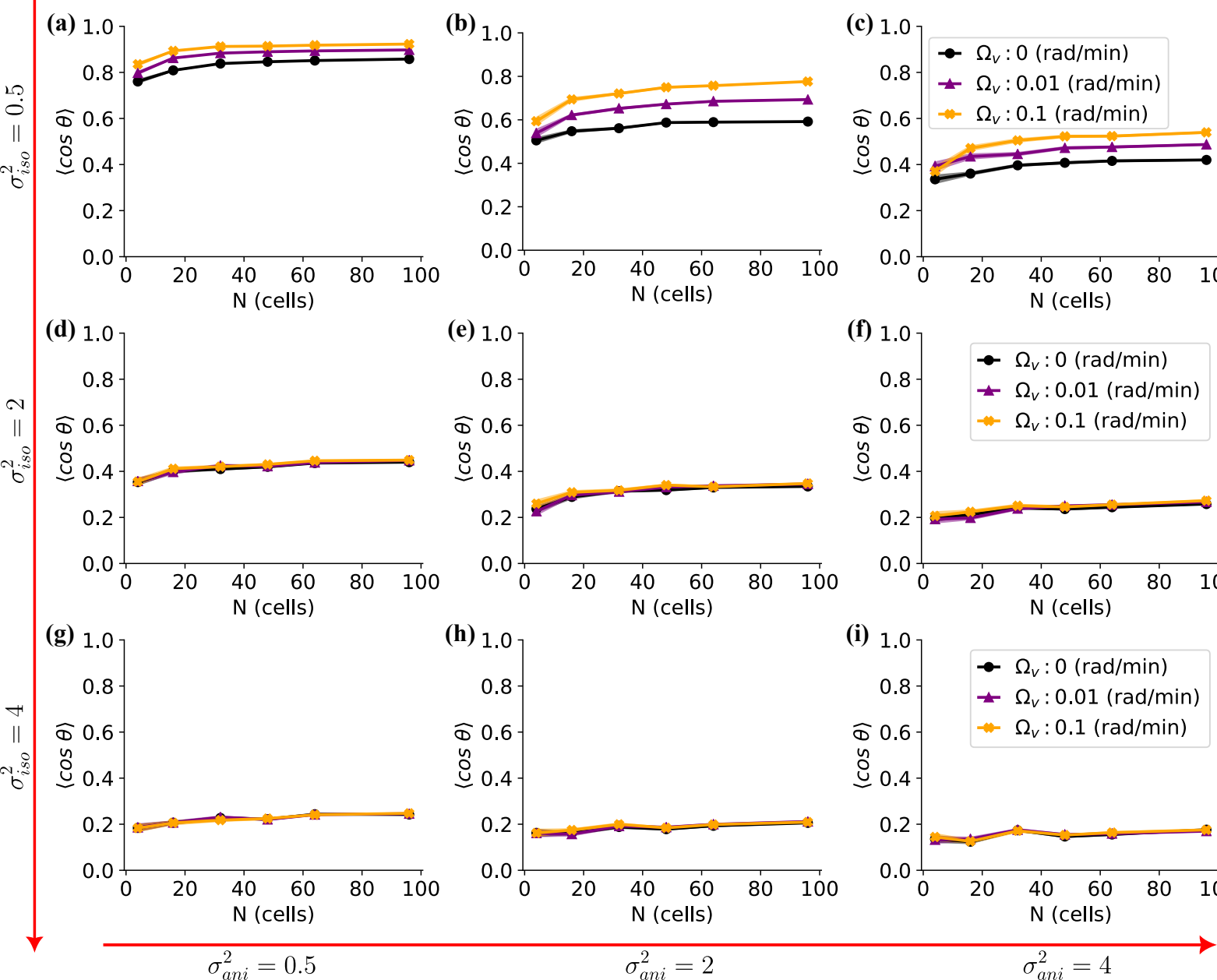

Supplement: S2 Fig — This plot is the same as Fig 1 but with a cell-cell interaction strength of k = 0.05 min−1. Isotropic component (σiso2) changes across rows (top to bottom) and anisotropic component (σani2) across rows (left to right) with specific values shown at right side and bottom of the figure (i.e. Figure (f) show directionalities for σiso2=2,σani2=4 ). The averages are over 40 simulations and each simulation is performed with 64 cells. For each simulation the reported directionality is the steady state average over final 5 hours of simulation Fig 1f. Results for different vaues of alignment rates to average velocity are color coded Ωv=0 rad/min (black), Ωv=0.01 rad/min (purple), and Ωv=0.1 rad/min (orange). The averaging time T for velocity is set to 1h. The shaded areas represent standard errors, although they may not be easily discernible due to their small size. (TIF) [file pone.0325800.s003.pdf]

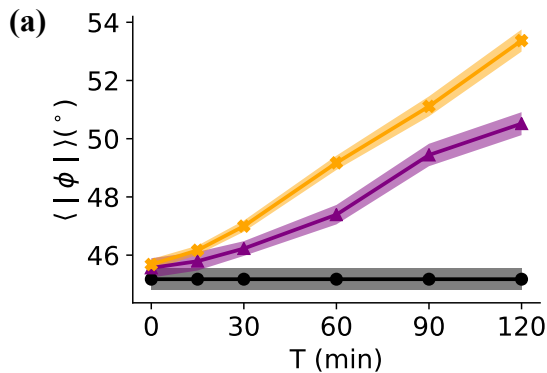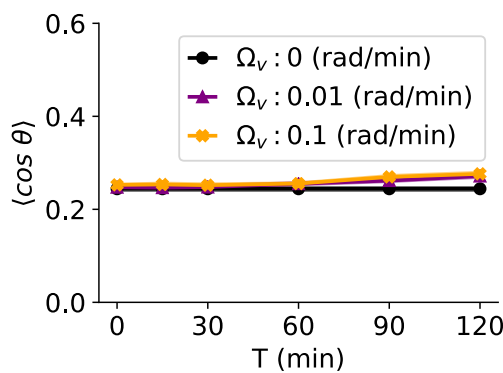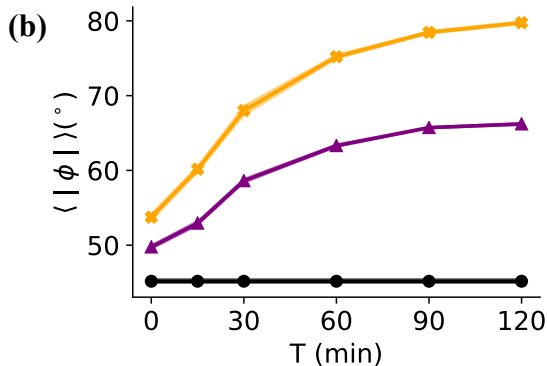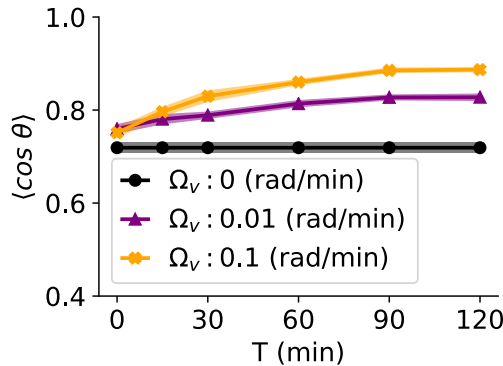

Supplement: S3 Fig — The reported values represent averages across 40 simulations of 64 cells with σiso2=2 & σani2=4 conducted at an interaction strength of (a) k = 0.05 min−1 and (b) k = 1 min−1. In the left column, the absolute value of the cell alignment angle is presented, while the right column displays the corresponding directionality. The shaded areas represent standard errors of the mean. (TIF) [file pone.0325800.s004.pdf]

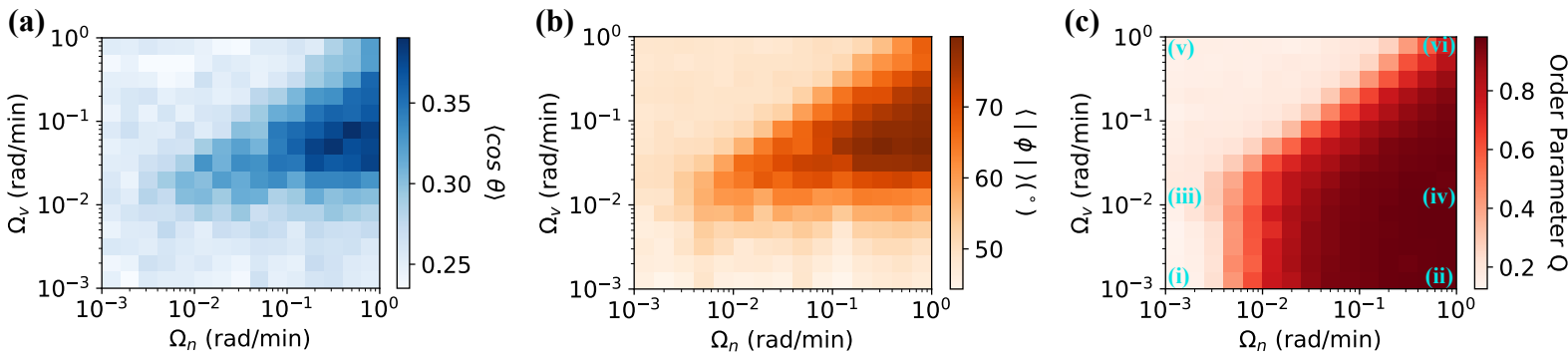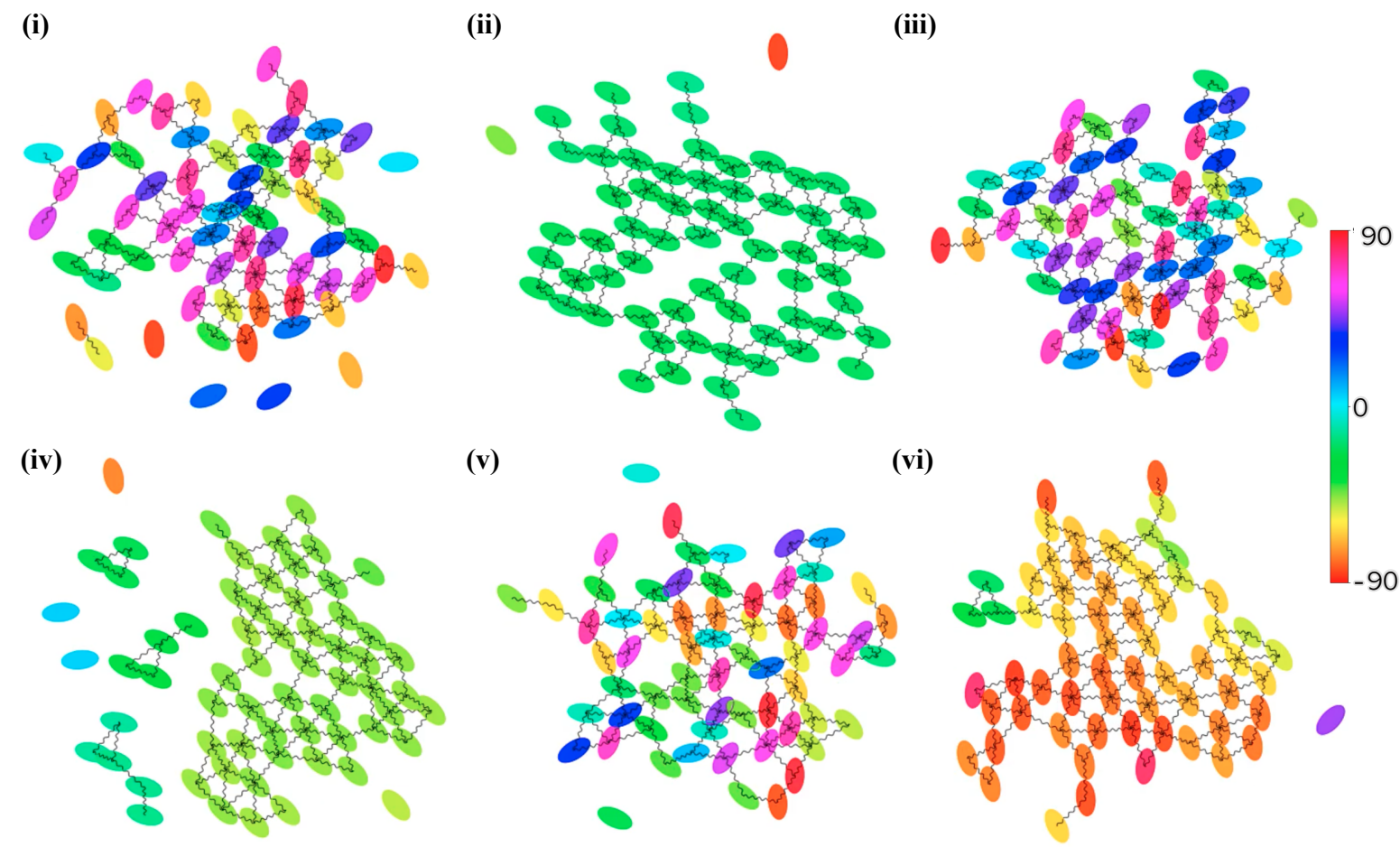

Supplement: S4 Fig — Each grid value represents an average result over 40 simulations conducted with 64 cells at the interaction strength of k = 0.05 min−1 with an averaging time T = 1 h with colorbars indicating corresponding numeric values. Example simulation snapshots for alignment rate tuples of (i), Ωv=0.001 rad/min, Ωn=0.001 rad/min; (ii), Ωv=0.001 rad/min, Ωn=1 rad/min; (iii), Ωv=0.012 rad/min, Ωn=0.001 rad/min; (iv), Ωv=0.012 rad/min, Ωn=1 rad/min; (v), Ωv=1 rad/min, Ωn=0.001 rad/min; (vi), Ωv=1 rad/min, Ωn=1 rad/min, also shown in panel c. Cells are colored according to their orientation shown on the colorbar. For all simulations σiso2=2 and σani2=4. (TIF) [file pone.0325800.s005.pdf]

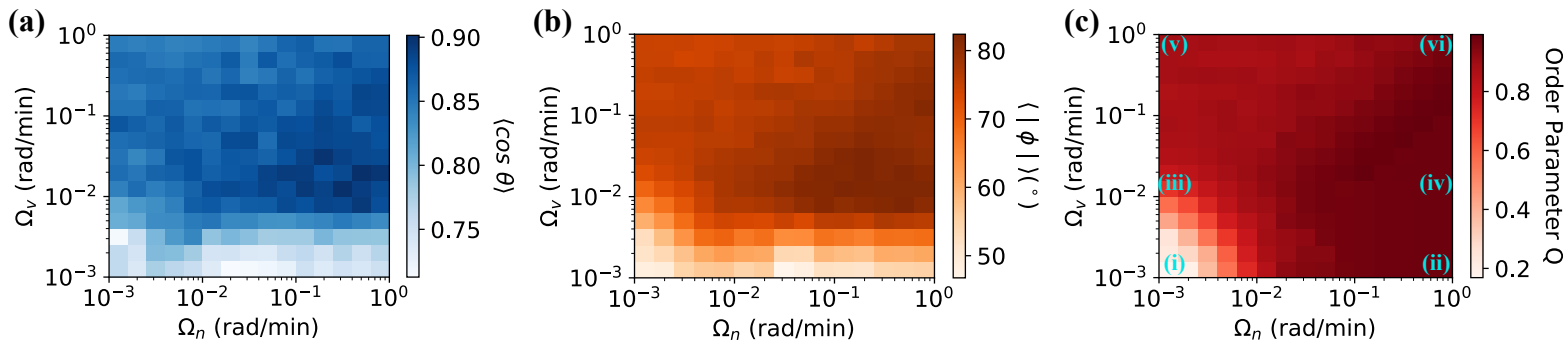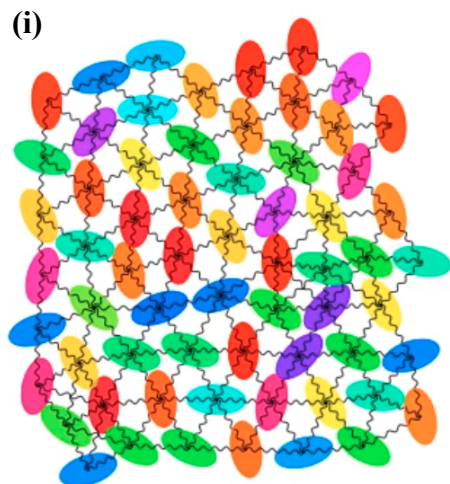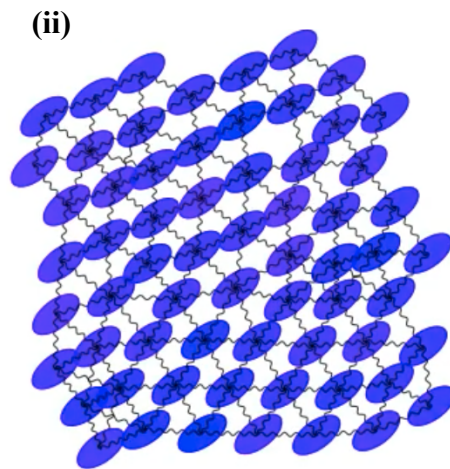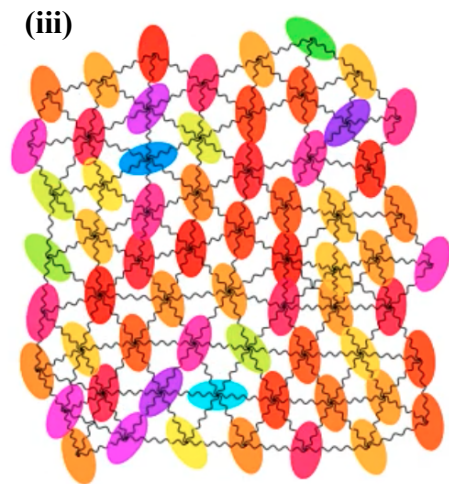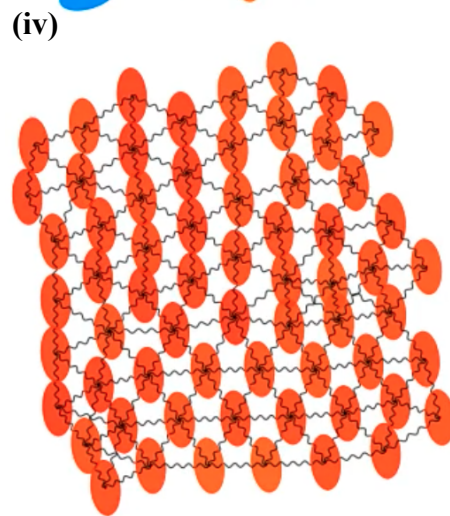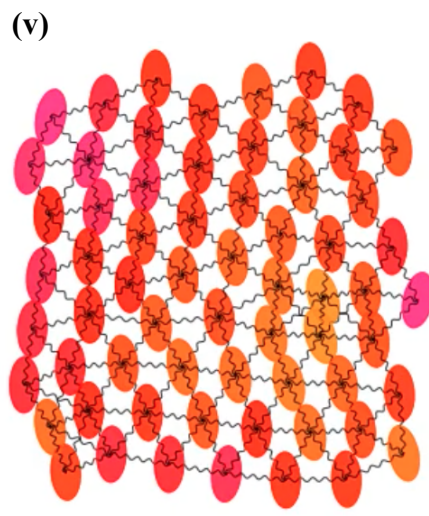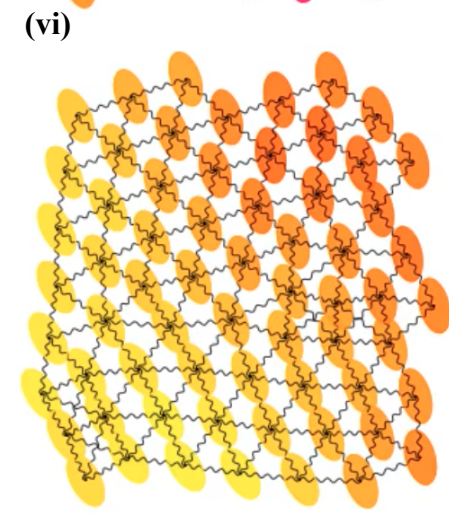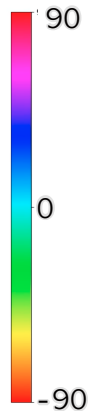

Supplement: S5 Fig — Each grid value represents an average result over 40 simulations conducted with 64 cells at the interaction strength of k = 1 min−1 with an averaging time T = 1 h with colorbars indicating corresponding numeric values. Example simulation snapshots for alignment rate tuples of (i), Ωv=0.001 rad/min, Ωn=0.001 rad/min; (ii), Ωv=0.001 rad/min, Ωn=1 rad/min; (iii), Ωv=0.012 rad/min, Ωn=0.001 rad/min; (iv), Ωv=0.012 rad/min, Ωn=1 rad/min; (v), Ωv=1 rad/min, Ωn=0.001 rad/min; (vi), Ωv=1 rad/min, Ωn=1 rad/min, also shown in panel c. Cells are colored according to their orientation shown on the colorbar. For all simulations σiso2=2 and σani2=4. (TIF) [file pone.0325800.s006.pdf]

**(a)**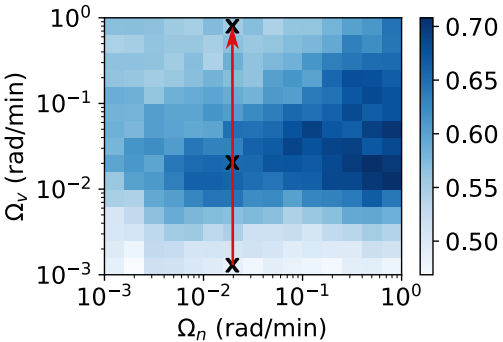**(b)**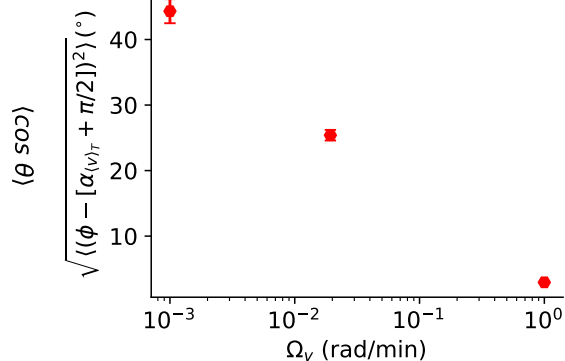**(c)**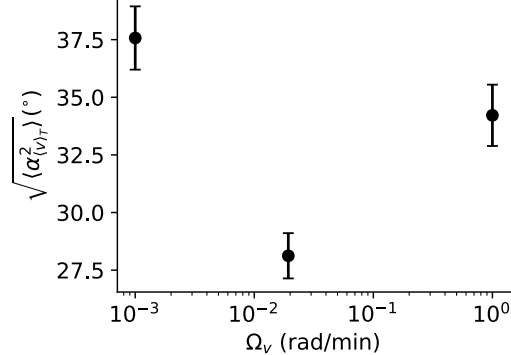

Supplement: S6 Fig — Panel (a) is the copy of Fig 4a with crosses showing where the exact measurement locations for plots in figures (b) and (c). (b) Root mean square deviation of cellular orientation ϕ from the direction of orthogonal to time averaged velocity α⟨vi⟩T+π/2 for different alignment rates Ωv. (b) Root mean square deviation of the direction of orthogonal to time averaged velocity α⟨vi⟩T+π/2 from favorable vertical π/2 orientation for different alignment rates Ωv. Each value in panels (b) and (c) represent an average over 40 simulations conducted with 64 cells that have isotropic and anisotropic variance components of σiso2=2 & σani2=4 at the interaction strength of k = 0.2 min−1 with an averaging time T = 1 h. Error bars indicate standard errors. (TIF) [file pone.0325800.s007.pdf]

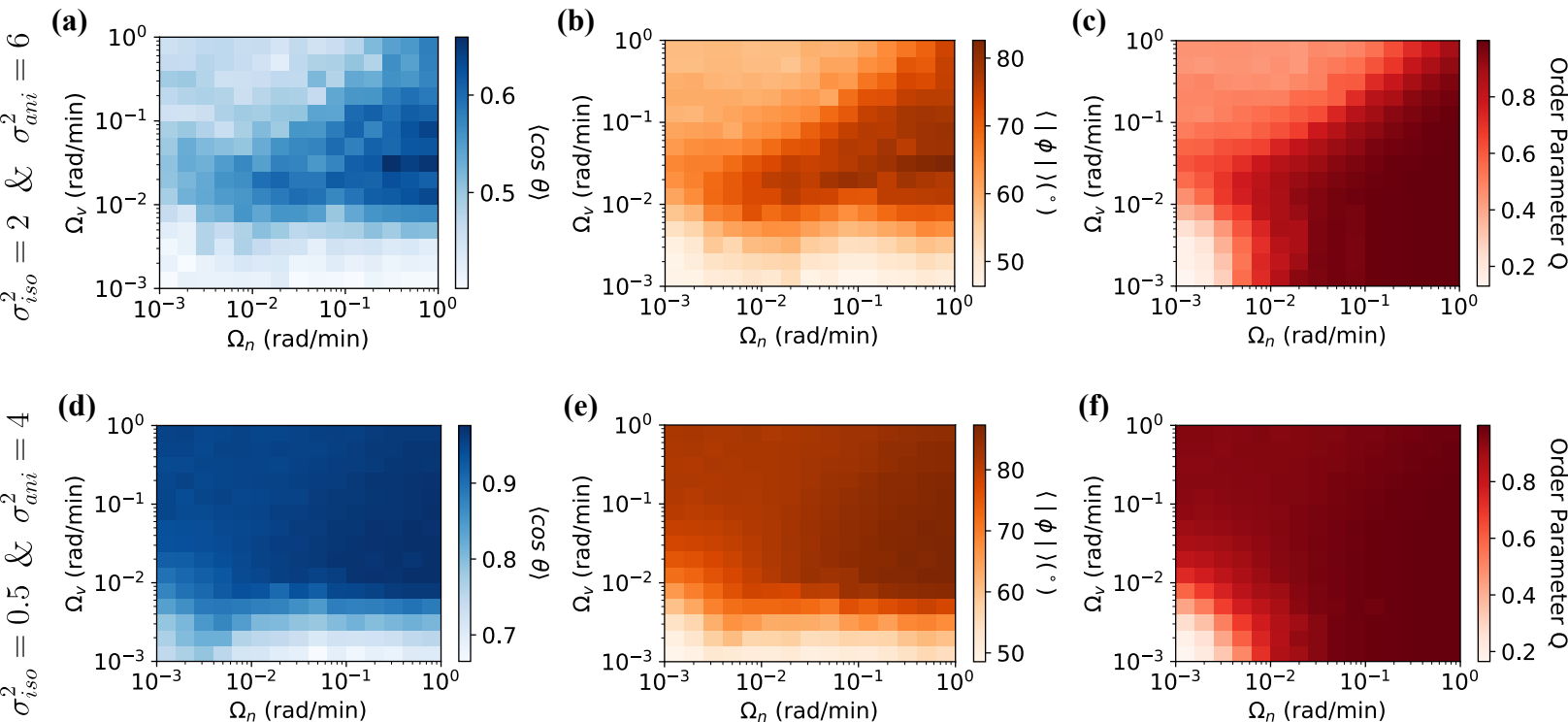

Supplement: S7 Fig — Phase diagrams on the top row show simulations for cells with isotropic and anisotropic components of variance of σiso2=2 & σani2=6, and bottom row represents simulations for σiso2=0.5 & σani2=4 Each grid value represents an average result over 40 simulations conducted with 64 cells at the interaction strength of k = 0.2 min−1 with an averaging time T = 1 h with colorbars indicating corresponding numeric values. (TIF) [file pone.0325800.s008.pdf]

**(a)**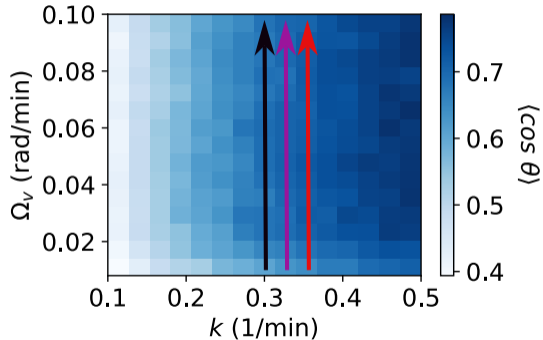**(b)**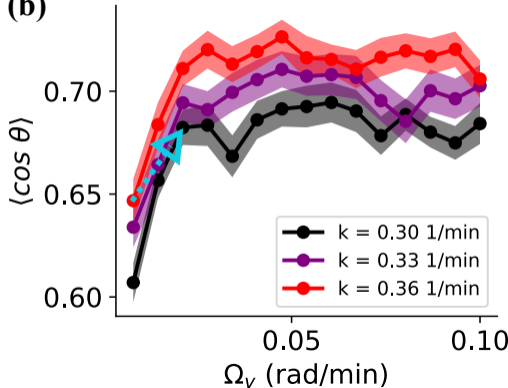

Supplement: S8 Fig — (a) Replication of the Fig 5c, left with arrows showing the spring constants used while varying Ωv for the plot on panel (b). Colors of the arrows correspond to the colors on the panel (b). (b) Directionality as a function of alignment rate to velocity for different values of interaction strength k. Even though decreasing adhesion strength generally results lower directionalities, the dotted cyan arrow shows a possible path of increase in cell orientation rate as adhesion decreases that could lead to higher directionality. The reported values represent averages across 40 simulations with 64 cells with σiso2=2 and σani2=4. The shaded areas represent standard errors of the mean. (TIF) [file pone.0325800.s009.pdf]

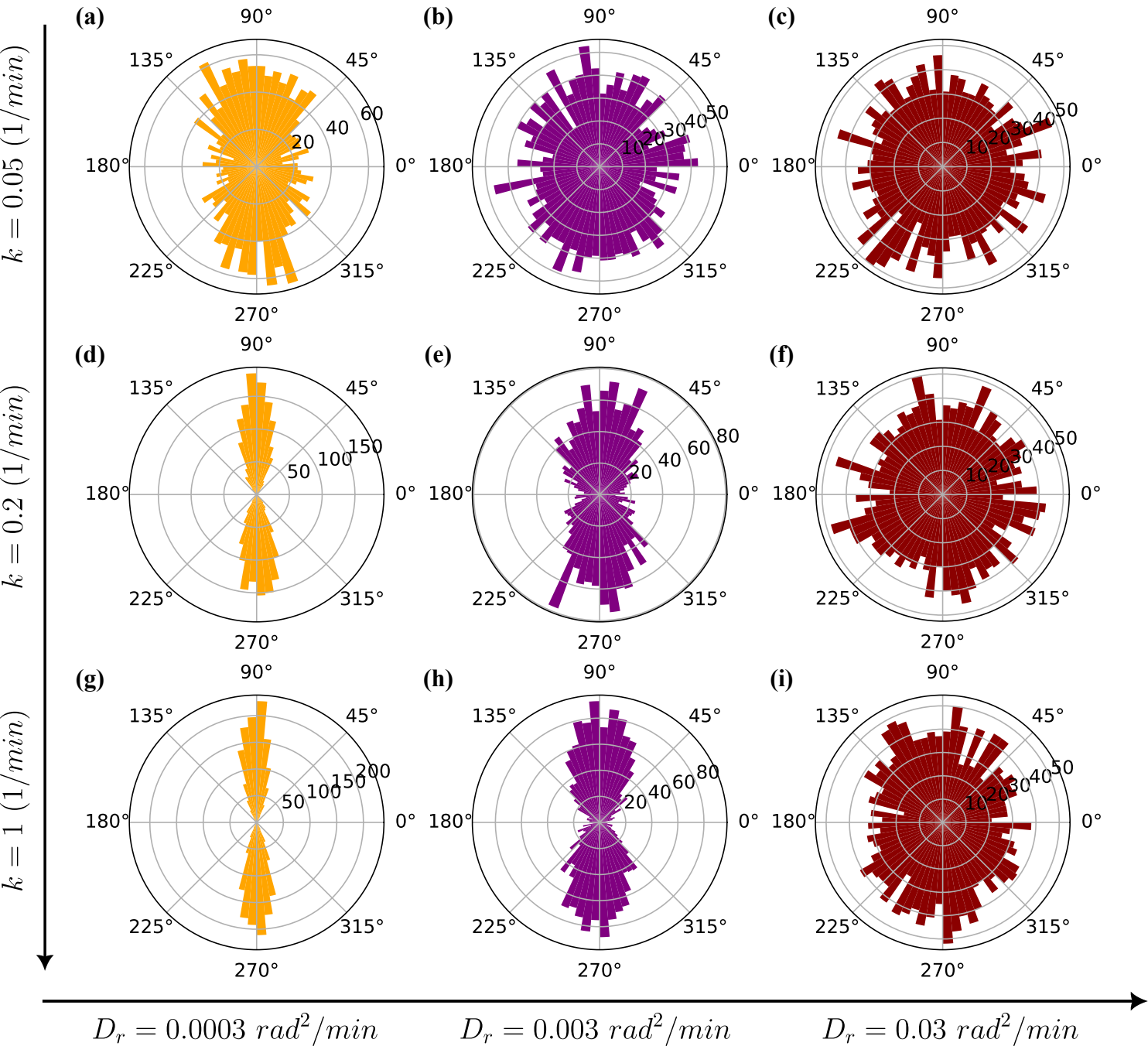

Supplement: S9 Fig — Interaction strength varies across rows (top to bottom) and diffusion coefficient across columns (left to right) with specific values of spring constant shown at right side and diffusion coefficient at bottom of the figure (i.e. panel (e) shows orientations for k=0.2 min−1 and Dr=0.003 rad2/min ). Each histogram is compiled from the final snapshot data of 40 simulations, each featuring 64 cells. Cells do not align to neighbors Ωn=0 rad/min, align to average velocity (averaging time T = 1 h) at the rate of Ωv=0.01 rad/min, and have σiso2=2 and σani2=4. (TIF) [file pone.0325800.s010.pdf]
